# The Immune‐Autonomic Interface in Aging: Baseline Immune Profile Shapes Cardiac Autonomic Response to Exercise

**DOI:** 10.1111/acel.70428

**Published:** 2026-03-06

**Authors:** Matías Castillo‐Aguilar, Lindybeth Sarmiento Varón, Carolina Pérez, Roberto Uribe‐Paredes, Marcelo A. Navarrete, Cristian Nuñez‐Espinosa

**Affiliations:** ^1^ Centro Asistencial Docente e Investigación (CADI), Universidad de Magallanes Punta Arenas Chile; ^2^ Escuela de Medicina, Universidad de Magallanes Punta Arenas Chile; ^3^ Departamento de Ingeniería en Computación Universidad de Magallanes Punta Arenas Chile; ^4^ Centre for Biotechnology and Bioengineering, CeBiB, Universidad de Chile Santiago Chile

**Keywords:** aging, autonomic nervous system, exercise, heart rate variability, immunosenescence, lymphocyte, muscle mass, resilience

## Abstract

Aging is characterized by reduced physiological resilience, linked to declines in both cardiac autonomic control (assessed via Heart Rate Variability, HRV) and immune function (immunosenescence, inflammaging). While static immune‐autonomic links are known, how baseline immune status dynamically influences autonomic responses to acute stress in aging remains unclear. This study investigated the association between baseline immune cell profiles and dynamic HRV changes during rest, acute exercise, and recovery in older adults. We quantified baseline lymphocyte subsets and assessed HRV during an exercise test. Using Bayesian mixed‐effects models, we found that while exercise significantly altered HRV as expected, baseline levels of specific immune cell subsets (e.g., B‐cells, T‐cells, CD4+/CD8+ ratio, and NK cells) were significantly associated with the pattern and magnitude of exercise‐induced HRV changes. This indicates that the pre‐existing immune state modulates the dynamic cardiac autonomic response to stress. Our findings highlight the critical role of immune‐autonomic crosstalk in shaping physiological resilience in aging, offering insights into heterogeneity in exercise responses and suggesting potential avenues for personalized health strategies.

## Graphical abstract

In 83 older adults, baseline lymphocyte phenotypes quantified by flow cytometry predicted the magnitude and direction of HRV shifts during a 2‐min step test and recovery. Bayesian models show that the immune landscape shapes exercise autonomic trajectories in a domain‐specific manner, revealing heterogeneity in physiological resilience.

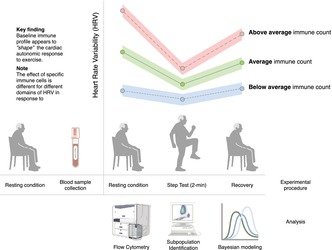

## 1 Introduction

The global demographic shift towards an aging population poses both a significant challenge and a unique opportunity for health science (Garmany et al. 2021; Bishop et al. 2022). While increased life expectancy is a major success of modern medicine, aging is, nonetheless, accompanied by a progressive decline in physiological function across multiple organ systems, leading to reduced resilience, increased vulnerability to stressors, and a higher burden of chronic diseases (Hotta and Uchida 2010; Reis et al. 2024; Teodoro et al. 2025).

Understanding the factors contributing to physiological resilience in aging is crucial; a cornerstone is the dynamic regulation exerted by the Autonomic Nervous System (ANS). The ANS orchestrates rapid, involuntary homeostatic adjustments across multiple systems (e.g., cardiovascular and respiratory), mediating responses to diverse challenges like exercise and stress (Castro et al. 2024; Castillo‐Aguilar et al. 2025). Cardiac autonomic control can readily be assessed through noninvasive measures such as Heart Rate Variability (HRV) and offers critical insights into autonomic function and systemic adaptability (Castillo‐Aguilar et al. 2023). HRV metrics reflect the interplay between sympathetic and parasympathetic branches that innervate the heart (Mosley and Laborde 2024). High HRV indicates robust parasympathetic tone and flexible regulation, associated with better health outcomes, including reduced cardiovascular morbidity and mortality (Olivieri et al. 2024). Conversely, reduced HRV is a hallmark of autonomic dysfunction, related to pathological states and diminished physiological reserve (Hotta and Uchida 2010; Olivieri et al. 2024). Aging is associated with a significant decline in HRV and an altered sympathovagal balance, contributing to reduced cardiovascular adaptability and stress resilience (Hotta and Uchida 2010; He et al. 2016; Olivieri et al. 2024). Exercise represents a fundamental physiological stressor acutely challenging autonomic control and requiring rapid, precise cardiac adjustments. HRV changes during and after exercise are vital indicators of autonomic responsiveness and recovery capacity (Olivieri et al. 2024; Castillo‐Aguilar et al. 2025).

Concurrently, the immune system undergoes profound age‐related changes collectively known as “immunosenescence” (Li et al. 2023; Reis et al. 2024; Bender et al. 2025). Immunosenescence is characterized by changes in leukocyte composition, dysregulated innate responses, impaired adaptive immunity, and notably a state of chronic low‐grade inflammation termed “inflammaging” (Li et al. 2023; Bender et al. 2025). Inflammaging, characterized by elevated systemic inflammatory mediators (e.g., cytokines), is strongly implicated in age‐related chronic diseases (Li et al. 2023; Bender et al. 2025). Changes in specific immune cell populations, such as lymphocyte subsets (T‐cells, B‐cells, and Natural Killer (NK) cells), have been associated with immunosenescence and inflammaging (Li et al. 2023; Reis et al. 2024; Bender et al. 2025; Teodoro et al. 2025). Aging is accompanied by quantitative and qualitative alterations in T‐cell compartments. Total CD3+ T‐cell counts (total T lymphocytes) tend to decline, whereas absolute CD8+ cytotoxic T‐cells numbers often remain relatively stable but become enriched for highly differentiated effector and terminally differentiated cells, partly driven by chronic antigenic exposure such as cytomegalovirus infection (Strindhall et al. 2013). These shifts are reflected in a progressive reduction of the ratio of CD4+ helper to CD8+ cytotoxic T cells (CD4:CD8), and an inverted ratio (< 1) has been proposed as a hallmark of the “immune risk profile” and a marker of immunosenescence and inflammaging (Strindhall et al. 2013; Serrano‐Villar et al. 2014). B‐cells also show a steady decline, resulting in decreased humoral immunity and poorer antibody responses (Sun et al. 2022). On the other hand, aging is associated with a shift towards a higher proportion of CD56‐dim NK cells, which are more cytotoxic and secrete fewer regulatory cytokines, and a relative decline in CD56‐bright NK cells, which exert stronger immunomodulatory functions (Solana et al. 2014; Khanmammadova et al. 2023; Rodriguez‐Mogeda et al. 2024).

It is increasingly recognized that the ANS and immune system are not isolated but engage in complex, bidirectional communication, forming a crucial neuro‐immune axis influencing systemic homeostasis (Lamotte et al. 2021; Jin et al. 2024; Wheeler and Quintana 2025). The ANS can modulate immune function, for example, via sympathetic innervation of lymphoid organs or the vagus nerve's “inflammatory reflex” (Tracey 2002; Jin et al. 2024; Wheeler and Quintana 2025). Conversely, immune cells and products (cytokines) can directly or indirectly influence autonomic neural activity. Pro‐inflammatory cytokines modulate autonomic activity by activating afferent pathways such as the vagus nerve, altering central autonomic network function, and modifying peripheral neurotransmitter release and receptor expression (Jin et al. 2024; Wheeler and Quintana 2025). Current understanding of this crosstalk, particularly in humans, stems mainly from studies of static associations in resting states, inflammatory conditions, or experimental manipulations (often animal/in vitro) (Halder and Lal 2021; Wu et al. 2021; Udit et al. 2022). These studies show correlations of immune activation or chronic inflammation with altered baseline autonomic tone or impaired reflexes.

Despite growing understanding of the immune‐autonomic axis, a knowledge gap remains in understanding how these systems interact dynamically, particularly under acute physiological stress. Specifically, it remains unclear whether an individual's baseline immune status influences autonomic adjustments required during and immediately after exercise in aging. Exercise is a potent modulator of both responses, and baseline state could theoretically influence the other's response. For instance, a pro‐inflammatory baseline profile (e.g., inflammaging) could impair rapid autonomic recovery or exacerbate sympathoexcitation during exercise. Conversely, a healthier immune profile might support more efficient regulation. Understanding this crosstalk has implications for explaining heterogeneity in aging exercise responses, identifying individuals at higher cardiovascular risk during stress, and informing the development of personalized interventions. Thus, this study investigated the relationship between baseline immune profiles and dynamic changes in cardiac autonomic modulation (assessed via HRV) during rest, acute exercise, and post‐exercise recovery in older adults. We hypothesized that specific baseline immune phenotypes would be associated with distinct exercise‐induced cardiac autonomic response and recovery patterns, indicating that the baseline immune state actively shapes autonomic control during stress in aging.

## 2 Material and Methods

### 2.1 Study Design

This investigation used an observational, correlational, and cross‐sectional design to explore how the baseline immune profile is linked with autonomic function. Given its cross‐sectional nature, data were collected at a single time, allowing for the examination of relationships between variables.

Before data collection began, all participants were thoroughly briefed on the objectives, procedures, and potential implications of the study. Informed consent was obtained from every participant to ensure ethical integrity and respect for individual autonomy.

### 2.2 Setting

This study was conducted at the Centro Asistencial Docente y de Investigación (CADI‐UMAG), an academic healthcare and research facility affiliated with the University of Magallanes, located in Punta Arenas, Chile. All assessments were conducted in a controlled environment between 9:00 and 11:00 a.m. to minimize the influence of circadian variability on physiological measurements.

The evaluation room was kept at a constant temperature of 20°C to ensure participant comfort and maintain standardized testing conditions, thereby reducing potential thermoregulatory effects on autonomic responses. Artificial white lighting was used to provide consistent illumination, preventing fluctuations in ambient light that could affect visual or cognitive factors during testing. Additionally, all assessments were conducted in a private and quiet setting to minimize external disturbances and ensure the reliability of the collected data.

### 2.3 Participants

Participants were recruited from the local community through advertisements and outreach efforts. A total of 83 older adults participated in the study, including 57 women and 26 men, with a mean age of 70.7 (SD = 5.8, range 61 to 89). To be eligible for inclusion, individuals had to meet the following criteria: (i) be 60 years of age or older at the time of enrollment; (ii) have permanent residency in the Magallanes and Chilean Antarctic region, ensuring a homogeneous population exposed to similar environmental and socioeconomic conditions; (iii) attain a score exceeding 60% on the Karnofsky Performance Status scale, a widely used measure of functional capacity, which ensured that participants had sufficient autonomy to perform the required study assessments (Schag et al. 1984); and (iv) have no prior diagnosis of conditions that could confound autonomic or cardiovascular function, including diabetic neuropathy, pacemaker implantation, clinical depression, cognitive impairment, motor disability, or dementia.

Exclusion criteria were established to minimize confounding factors that could influence autonomic measurements. Participants were excluded if they (i) were using beta‐blockers during the study period, as these medications can significantly alter autonomic and cardiovascular responses; (ii) had consumed any stimulant substances, including caffeine or medications with sympathomimetic effects, within 12 h before cardiac assessment; or (iii) had any degree of motor impairment that restricted independent movement and could interfere with study procedures. Notably, no recruited participants met the exclusion criteria.

### 2.4 Procedures

Participants attended a single study visit after a 12‐h fast and refrained from strenuous exercise and alcohol consumption. Upon arrival, participants provided informed consent and underwent a brief health screening to double‐check eligibility based on inclusion/exclusion criteria, including blood pressure assessment (SBP < 140 mmHg and DBP < 90 mmHg). While 130/80 mmHg is the diagnostic threshold for hypertension, the more conservative 140/90 mmHg cutoff is widely used to balance safety with participant inclusion, and thus was appropriate for our study design.

Sociodemographic data were collected via structured interview, including name, age, sex, marital status, educational attainment, and existing chronic medical conditions. This information served to characterize the sample and identify potential confounding factors.

Following the initial registry, body composition measurements were performed using multi‐frequency bioelectrical impedance analysis (BIA) according to standardized procedures. Participants were asked to empty their bladders immediately before this assessment.

A peripheral venous blood sample (4 mL) was collected in EDTA tubes by a trained nurse. Samples were gently homogenized and maintained at room temperature for subsequent immunophenotypic analysis and processed within 2 h of collection.

After a minimum 10‐min rest period in a seated position, participants were fitted with a Polar H10 heart rate monitor for continuous R‐R interval recording and an Omron Hem‐7142 monitor for blood pressure measurements. Following baseline physiological measurements (HRV segment from rest and blood pressure), participants performed the Two‐Minute Step Test (TMST) as an acute physiological stressor. R‐R intervals were recorded continuously throughout the 2‐min test and for a 5‐min recovery period immediately following the test. Blood pressure was measured again immediately upon completion of the TMST to ensure the safety of participants.

The sequence of functional capacity tests (Chair‐Sit‐to‐Stand, Timed Up‐and‐Go) was conducted after the TMST and recovery period, ensuring adequate rest between assessments to avoid carry‐over fatigue.

### 2.5 Assessments

#### 2.5.1 Two‐Minute Step Test (TMST)

The Two‐Minute Step Test (TMST), a standardized protocol from the Senior Fitness Test battery (Rikli and Jones 1999), was employed as an acute exercise‐induced physiological stressor to challenge cardiac autonomic control. While also providing an index of lower body endurance and functional mobility, the primary purpose of the TMST in this study was to elicit a controlled, submaximal physiological perturbation suitable for assessing dynamic autonomic responses in older adults. Participants were instructed to march in place for 2 min, lifting their knees to a height midway between the patella and the iliac crest, guided by verbal cues. The total number of steps completed served as a measure of the exercise volume and functional performance.

The total number of steps completed in 2 min was recorded as the outcome measure for functional capacity, while the physiological stress induced by this standardized exertion served as the basis for assessing dynamic autonomic responses.

#### 2.5.2 Chair‐Sit‐To‐Stand (CSTS) Test

Lower body strength and power were assessed using the 30‐s Chair‐Sit‐to‐Stand (CSTS) Test, also part of the Senior Fitness Test battery (Rikli and Jones 1999). Participants were seated on a standard chair (approximately 17 in. high) with their feet flat on the floor and arms crossed over their chest. They were instructed to stand up completely and then sit back down as many times as possible within 30 s. The outcome measure was the total number of stands completed in 30 s.

#### 2.5.3 Timed Up‐and‐Go (TUG) Test

Functional mobility, including balance and gait speed, was assessed using the Timed Up‐and‐Go (TUG) test (Rikli and Jones 1999). Participants were seated in a standard armchair. Upon the command “Go”, they stood up from the chair, walked a distance of 3 m, turned around, walked back to the chair, and sat down. The time taken to complete the task was recorded in seconds using a stopwatch, starting from the command “Go” and stopping when the participant was fully seated back in the chair. The average time of the two‐timed trials was used as the outcome measure.

#### 2.5.4 Body Composition

Body composition, including body mass index (BMI), body fat percentage, lean muscle mass, body water, and bone mass, was assessed using multifrequency bioelectrical impedance analysis (BIA). Measurements were performed using the Tanita BC‐558 Iron‐man Seg‐mental Body Composition Monitor (Tanita Ironman, Ar‐lington Heights, IL 60005 USA). Participants were instructed to fast for at least 4 h prior to the assessment and to avoid strenuous exercise and alcohol consumption for at least 12 h. They were also asked to empty their bladder before the measurement. Participants stood barefoot on the device's foot electrodes and held the hand electrodes while wearing light clothing.

#### 2.5.5 Immune Profiling

Peripheral blood was collected into 4 mL EDTA tubes and kept at room temperature. Samples were gently homogenized and processed within 2 h of collection to preserve cell integrity. Immunophenotyping was performed by flow cytometry on a CytoFLEX S instrument (Beckman Coulter) equipped with four lasers, allowing up to 13 parameters to be measured in a single acquisition. A custom panel of BioLegend monoclonal antibodies was used to distinguish major lymphocyte subsets: CD45 (Brilliant Violet 570), CD3 (APC/Cy7), CD56 (PE), CD8 (Alexa Fluor 700), CD4 (APC), and CD19 (FITC). Each antibody was titrated to determine the optimal staining concentration, and compensation was set using single‐stained controls to correct for spectral overlap.

For staining, 100 μL aliquots of whole blood (1 × 10^6^ cells) were incubated with the antibody cocktail for 20 min at room temperature in the dark. Red blood cells were lysed by adding 125 μL of OptiLyse C (Beckman Coulter), mixing briefly and incubating for a further 10 min in the dark. After lysis, 1 mL of IsoFlow sheath fluid (Beckman Coulter) was added; samples were gently mixed and acquired at a medium flow rate, recording ≥ 30,000 total events per sample. Absolute counts (cells/μL) were obtained from the CytoFLEX S events/μL parameter (volumetric counting), derived from the instrument‐estimated sample volume acquired; dilution factors were applied when applicable. Data were analyzed using FlowJo software (v10.10.0; BD Life Sciences).

Analyses were performed using a hierarchical gating strategy (singlets → cells → CD45+ lymphocytes), as detailed in the revised gating‐strategy figure (Figure S1A). Within the CD45+ lymphocyte gate, T lymphocytes (T cells) were identified as CD3+ events and further subdivided on a CD4 versus CD8 dot plot into CD3+CD4+CD8‐ helper T cells (CD4+ T cells), CD3+CD4‐CD8+ cytotoxic T cells (CD8+ T cells), CD3+CD4+CD8+ (double‐positive T cells), and CD3+CD4‐CD8‐ (double‐negative/other T cells). These T‐cell subsets were expressed as percentages of CD3+ T cells and included in the main dataset (Figure S1B). Within the CD45+ lymphocyte gate, natural killer (NK) cells were identified as CD3‐CD56+ events on a CD3 versus CD56 plot. CD56 expression among CD3‐CD56+ cells was then displayed on a one‐dimensional histogram, and CD56^bright^ and CD56^dim^ NK‐cells subset were defined as populations with higher and lower CD56 fluorescence intensity, respectively, within this CD3‐CD56+ gate. CD3 + CD56‐ T cells from the same sample were used as an internal CD56‐ reference to position the CD56+ threshold, as illustrated in Figure S1C.

##### 2.5.5.1 Extended Regulatory and Memory Lymphocyte Phenotyping

In a subset of participants with cryopreserved peripheral blood mononuclear cells (PBMCs; *n* = 9), we performed extended flow‐cytometry panels to characterize regulatory and memory lymphocyte populations, including regulatory T cells (Tregs), memory B cells (Bmem) with CD21^low^ subsets (B_mem/CD21^low^), and regulatory/transitional B cells (Bregs). PBMCs were thawed, washed, and resuspended in 1% BSA/PBS, split into an unstained tube and three staining tubes, incubated with antibodies for 20 min at 4°C in the dark, washed, and acquired on the same CytoFLEX S instrument. The unstained tube was used to define negative populations and guide the gate boundary. Data were analyzed using a hierarchical gating strategy (singlets → cells → viable cells → CD45+ lymphocytes), detailed in Figure S2A.

The regulatory T‐cells (Treg) panel included 7‐AAD (viability), CD45 (BV570), CD3 (APC/Cy7), CD4 (APC), CD25 (FITC), and CD127 (AF700). After gating singlets → cells → viable cells (7‐AAD‐) → CD45+ lymphocytes → CD3+CD4+ T cells, Tregs were defined as CD3+CD4+CD25^hiCD127^low and expressed as a percentage of CD4+ T cells (Figure S2B).

The memory B‐cell (Bmem) panel comprised 7‐AAD, CD45 (BV570), CD3 (FITC, dump channel), CD19 (ECD), CD27 (Pacific Blue), CD21 (APC/Fire750), and IgG (APC). Within CD45+ lymphocytes, B cells were defined as CD3‐CD19+, and the following subsets were quantified as percentages of total CD19+ cells: naïve B cells (CD19+CD27‐), total memory B cells (CD19+CD27+), IgG‐switched memory B cells (CD19+CD27+IgG+), and CD21^low^ B cells (CD19+CD21^low^) (Figure S2C).

The regulatory/transitional B‐cells (Breg) panel included 7‐AAD, CD45 (BV570), CD3 (FITC, dump), CD19 (ECD), CD24 (PE), CD38 (APC/Fire750), and IgM (Pacific Blue). After gating to CD3‐CD19+ B cells, putative regulatory/transitional B cells were defined as CD19+CD24^hi^ CD38^hi^, and their IgM expression profile was described within this gate (Figure S2D).

For each subject in the PBMC subsample, we calculated the percentage of Tregs, naïve and memory B cells (including IgG‐switched and CD21^low^ subsets), and Bregs. These frequencies (mean ± SD) are summarized in Table S2 and Figure S4. Because these extended phenotypes were obtained only in a subsample, they are reported descriptively and were not incorporated into the main Bayesian regression models.

#### 2.5.6 Cardiovascular Parameters

Systolic blood pressure (SBP) and diastolic blood pressure (DBP) were measured using an Omron pressure monitor to establish baseline cardiovascular status. To ensure participant safety and the reliability of HRV measurements, only individuals with SBP < 140 mmHg and DBP < 90 mmHg at the time of assessment were eligible for HRV recording.

Cardiac autonomic modulation was evaluated through continuous R‐R interval recordings obtained using the Polar Team2 system (Polar). Participants remained seated throughout the HRV measurement procedure, with R‐R intervals recorded continuously during the last 10 min of rest. From this data, a stable 5‐min segment was selected for analysis (Castillo‐Aguilar et al. 2023). Participants breathed spontaneously, and signal preprocessing included excluding artifacts and ectopic heartbeats, which did not exceed 3% of the recorded data (Malik 1996).

Time‐domain HRV parameters included the root mean square of successive R‐R interval differences (RMSSD, in ms), an established index of parasympathetic activity, and the standard deviation of R‐R intervals (SDNN), which reflects overall autonomic modulation, encompassing both sympathetic and parasympathetic contributions (Buchheit et al. 2010). In the frequency domain, high‐frequency (HF) power was analyzed as a marker of parasympathetic activity, including respiratory sinus arrhythmia. In contrast, low‐frequency (LF) power was assessed due to its association with baroreflex activity. Additionally, very low‐frequency (VLF) power was considered, given its reported relationship with emotional stress.

The Parasympathetic Nervous System Index (PNS Index) and Sympathetic Nervous System Index (SNS Index) were calculated to further quantify autonomic balance. The PNS Index, reflecting total vagal activity, was derived from mean R‐R intervals, RMSSD, and the Poincaré Plot SD_1_ index, all expressed in normalized units (Berntson et al. 1997; Rajendra Acharya et al. 2006). The SNS Index, indicative of sympathetic influence, was calculated using mean R‐R intervals, Baevsky's Stress Index (a measure of cardiovascular and autonomic stress), and the Poincaré Plot SD_2_ index (Berntson et al. 1997; Rajendra Acharya et al. 2006). The Stress Index (SI), a normalized measure derived from Baevsky's SI, was also included to indicate autonomic system load (Baevsky and Chernikova 2017).

All HRV analyses were conducted using Kubios HRV software (Kuopio, Finland).

### 2.6 Statistical Analysis

We adopted a fully Bayesian modeling approach to examine the confounding influences on cardiac autonomic modulation in response to exercise. This approach avoids several well‐known limitations of null‐hypothesis significance testing, including dichotomisation based on arbitrary *p* value thresholds and strong dependence on sample size. All dependent and independent variables were standardized (z‐scored) before analysis to facilitate interpretation across different scales and to enhance the efficiency of parameter estimation. Further model details, such as confounding adjustments, can be seen in the “Statistical analysis” section of Appendix S1.

Continuous variables are reported as the arithmetic mean ± standard deviation (mean ± SD), whereas categorical variables are expressed as absolute counts (*n*) and percentages (%).

Results follow the SEXIT (Sequential Effect eXistence and Significance Testing) framework (Makowski et al. 2019), reporting the posterior median and its 95% highest‐density credible interval, the probability of direction (pd) as a measure of effect existence. Practical significance is indexed by the proportion of posterior mass outside a region of practical equivalence (ROPE) defined as ±0.1 times the response variable's standard deviation (Makowski et al. 2019). Bayes factors (BF_10_) were reported and computed via the Savage–Dickey density ratio against the point null to provide an absolute measure of evidence for or against the null hypothesis (Heck 2019; Jeffreys 1998). As a rule of thumb, ps ≥ 95% and BF_10 > 1 are taken to indicate reasonably strong evidence for an association, while lower values are interpreted as weaker or more uncertain evidence. To ensure consistency in ROPE interpretations, all predictors, including age and questionnaire scores, were standardized prior to modeling. All analyses were implemented in R (R Core Team 2021).

## 3 Results

### 3.1 Sample Characteristics

The final included sample involved 83 older individuals, aged 70.7 (SD = 5.8, range 61–89) years old and average BMI of 30.4 (SD = 6.1, range 18.4–57.2) kg/m^2^, with 68.7% (*n* = 57) of the collected sample consisting of females (males, *n* = 26 [31.3%]). Further sample characteristics are displayed in Table 1.

**TABLE 1:** Sample characteristics in sociodemographical, anthropometric, and functional variables.

Characteristic	Overall *N* = 83	Sex		Difference	95% CI
		Male *N* = 26	Female *N* = 57		
Age	70.7 ± 5.8	71.8 ± 5.9	70.2 ± 5.7	0.28	−0.18, 0.75
Systolic BP (mm Hg)	132.6 ± 15.6	132.6 ± 13.7	132.6 ± 16.5	0.00	−0.47, 0.46
Diastolic BP (mm Hg)	79.1 ± 10.7	81.7 ± 11.3	77.9 ± 10.4	0.35	−0.11, 0.82
Weight (kg)	74.4 ± 14.5	79.0 ± 13.0	72.4 ± 14.7	0.48	0.00, 0.95
Height (cm)	156.6 ± 8.4	164.5 ± 7.4	153.0 ± 6.1	1.7	1.2, 2.3
BMI (kg/m^2^)	30.4 ± 6.1	29.1 ± 4.5	31.0 ± 6.7	−0.34	−0.82, 0.13
Body fat (%)	35.2 ± 8.8	26.1 ± 6.2	39.4 ± 6.4	−2.1	−2.7, −1.6
Muscle mass (kg)	45.2 ± 8.6	54.6 ± 7.4	40.9 ± 4.9	2.2	1.6, 2.8
Body water (%)	47.5 ± 7.6	54.4 ± 5.3	44.4 ± 6.4	1.7	1.2, 2.3
Bone mass (kg)	2.4 ± 0.4	2.9 ± 0.4	2.2 ± 0.2	2.3	1.7, 2.9
TMST steps (n)	91.4 ± 25.9	97.3 ± 26.6	88.6 ± 25.4	0.34	−0.13, 0.81
TUG (seg)	6.1 ± 1.9	5.7 ± 2.7	6.2 ± 1.4	−0.25	−0.71, 0.22
CSTS (reps)	16.3 ± 4.6	17.7 ± 4.8	15.7 ± 4.4	0.43	−0.04, 0.90

*Note:* Statistics are presented as [mean] ± [standard deviation]. “Difference” column denotes the standardized mean difference between males and females. CI, 95% confidence interval from the difference column.

Abbreviations: BMI, body mass index; BP, blood pressure; CSTS, chair sit‐to‐stand; TMST, two‐minute step test; TUG, timed‐up‐and‐go.

### 3.2 Exercise‐Induced Autonomic Response

Initially, autonomic signatures exhibited dynamic, time‐dependent alterations in response to exercise‐induced physiological strain throughout the different phases of the exercise protocol.

During the two‐minute step test, mean heart rate increased from approximately 70.14 ± 8.69 beats/min at rest to 102.80 ± 16.41 beats/min, consistent with a moderate‐to‐vigorous exercise intensity in this age group.

Analysis of time‐domain parameters revealed an immediate reduction in RMSSD (*β* = −1.1, 95% CI [−1.33, −0.88], pd = 100%, ps = 100%, BF_10_ = 6213.68), SDNN (*β* = −0.98, 95% CI [−1.22, −0.75], pd = 100%, ps = 100%, BF_10_ = 6403.31), and mean RRi (*β* = −1.62, 95% CI [−1.78, −1.45], pd = 100%, ps = 100%, BF_10_ = 4388.73) at the beginning of exercise, compared to resting values. Similarly, following exercise cessation, a sustained marked reduction in mean RRi was observed compared to baseline measurements (*β* = −0.4, 95% CI [−0.56, −0.24], pd = 100%, ps = 100%, BF_10_ = 604.38). However, this pattern was not observed for RMSSD and SDNN. RMSSD returned to baseline measurements (*β* = 0, 95% CI [−0.23, 0.22], pd = 48.8%, ps = 18.6%, BF_10_ = 0.04), whereas SDNN exhibited a marginal increase during recovery compared to baseline records (*β* = 0.27, 95% CI [0.04, 0.51], pd = 98.7%, ps = 91.8%, BF_10_ = 0.48).

Frequency domains experienced outcomes similar to those of time domains. A marked decrease was observed in all frequency domains during exercise compared to resting values. A greater decrease was observed in HF (*β* = −1.02, 95% CI [−1.25, −0.79], pd = 100%, ps = 100%, BF_10_ = 6340.75), followed by LF (*β* = −0.61, 95% CI [−0.89, −0.31], pd = 100%, ps = 100%, BF_10_ = 7889.24), and then VLF, which showed the smallest decrease of the three (*β* = −0.45, 95% CI [−0.75, −0.16], pd = 99.8%, ps = 98.9%, BF_10_ = 3.41). Despite HF and VLF returning to baseline values post‐exercise (HF, *β* = 0.07, 95% CI [−0.17, 0.3], pd = 71.1%, ps = 39%, BF_10_ = 0.05; VLF, *β* = 0.17, 95% CI [−0.13, 0.47], pd = 87.3%, ps = 68.6%, BF_10_ = 0.1), LF, conversely, increased post‐exercise relative to baseline measurements (*β* = 0.32, 95% CI [0.02, 0.6], pd = 98.6%, ps = 93.4%, BF_10_ = 0.51).

Finally, we analyzed the behavior of composite indices in response to exercise. In this domain, we observed a marked increase in the SNS index (*β* = 1.51, 95% CI [1.29, 1.72], pd = 100%, ps = 100%, BF_10_ = 5878.42) and the Stress index (*β* = 1.23, 95% CI [0.98, 1.48], pd = 100%, ps = 100%, BF_10_ = 6638.9) during exercise. This initial autonomic response was not observed in the PNS index (*β* = −0.08, 95% CI [−0.39, 0.25], pd = 68.8%, ps = 45.5%, BF_10_ = 0.06). When inspecting the post‐exercise recovery of these composite indices, all measures returned to comparable baseline values at post‐exercise recordings (PNS, *β* = −0.07, 95% CI [−0.39, 0.25], pd = 66.4%, ps = 42.1%, BF_10_ = 0.06; SNS, *β* = 0.1, 95% CI [−0.11, 0.31], pd = 81.9%, ps = 49.4%, BF_10_ = 0.06; Stress, *β* = −0.08, 95% CI [−0.34, 0.16], pd = 74.6%, ps = 44.6%, BF_10_ = 0.05).

**FIGURE 1:**
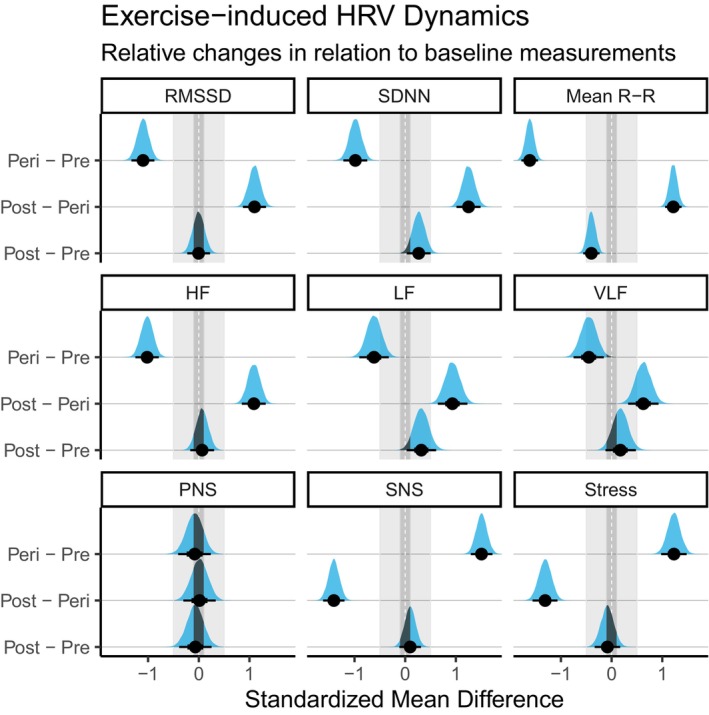
Posterior distribution of the pairwise time differences of exercise‐induced dynamics for HRV time‐, frequency‐domains and autonomic indices. “Peri‐Pre” represents the immediate response to exercise relative to baseline, “Post‐Peri” represents immediate post‐exercise recovery relative to in‐exercise measurements, and “Post‐Pre” represents autonomic recovery post‐exercise relative to baseline. White and dashed vertical line denotes the null effect. Shaded areas denote the range of practical equivalence (ROPE) and the area of medium effect at 0.5 standardized units. All effects are adjusted for confounders.

These effects, adjusted for confounding factors, are portrayed in Figure 1.

### 3.3 Autonomic‐Immune Axis

When assessing the bidirectional nature of the autonomic‐immune axis, we examined the relationship between measures of autonomic control and its dynamics related to exercise‐induced strain given by baseline immunological markers.

**FIGURE 2:**
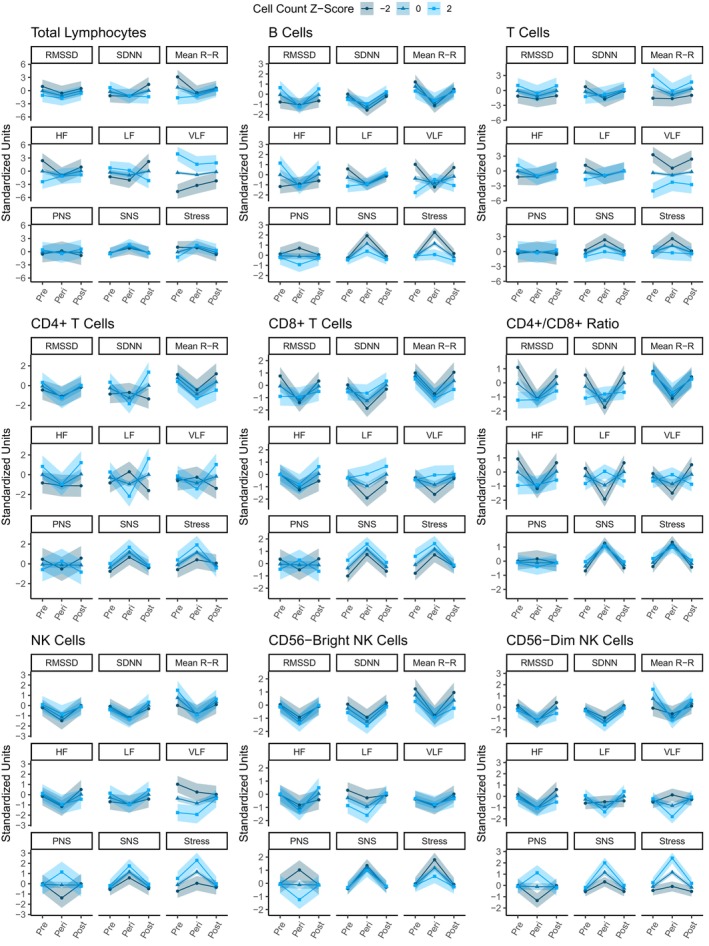
Predicted exercise‐induced cardiac autonomic modulation by standardized immune cell counts across discrete time frames. The trajectories illustrate the relative dynamics of HRV parameters in response to exercise, based on immune cell count groups standardized as z‐scores. “Pre” represents the baseline resting condition, “Peri” represents the in‐exercise HRV measurements, and “Post” represents the post‐exercise 5‐min recovery window. All depicted effects are adjusted for confounders.

Predicted exercise‐induced cardiac autonomic dynamics, stratified by standardized cell counts for each immune phenotype, are depicted in Figure 2.

#### 3.3.1 Total Lymphocytes

When examining the effect of baseline lymphocyte counts, we observed varying effects on cardiac autonomic trajectories in response to exercise‐induced strain.

Time‐domain parameters were mostly unaffected by variations in baseline lymphocyte counts. At baseline, we observed that greater immune cell quantities were associated with a 75% posterior probability of reduced RMSSD (*β* = −0.5, 95% CI [−1.98, 0.94], pd = 75%, ps = 70.4%, BF_10_ = 0.3), a 73.8% posterior probability of greater SDNN (*β* = 0.47, 95% CI [−0.93, 1.94], pd = 73.8%, ps = 69.3%, BF_10_ = 0.3), and a 95.1% posterior probability of reduced mean RR interval at rest (*β* = −1.2, 95% CI [−2.65, 0.25], pd = 95.1%, ps = 93.3%, BF_10_ = 0.91). Nonetheless, the effects of immune cell counts on resting time‐domain HRV differed from those observed during exercise. Specifically for mean RR, we observed a 94.4% posterior probability of an effect inversion during exercise, suggesting that greater lymphocyte counts were associated with reduced mean RR at rest but increased mean RR during exercise (interaction effect, *β* = 1.00, 95% CI [−0.21, 2.32], pd = 94.4%, ps = 92.3%, BF_10_ = 0.76).

Frequency domains exhibited patterns similar to those aforementioned, whereas total lymphocyte counts were observed to influence the spectral components of HRV at baseline and during exercise. HF was negatively associated with immune cell counts at rest (*β* = −1.2, 95% CI [−2.68, 0.38], pd = 93.6%, ps = 91.7%, BF_10_ = 0.81), but positively during exercise (interaction effect, *β* = 1.16, 95% CI [−0.59, 2.91], pd = 89.8%, ps = 87.9%, BF_10_ = 0.68). Although less pronounced, LF had a positive association with total lymphocyte counts at rest (with a 74.9% posterior probability; *β* = 0.51, 95% CI [−1.03, 1.96], pd = 74.9%, ps = 70.5%, BF_10_ = 0.32). A similar effect was observed for VLF at rest (*β* = 2.15, 95% CI [0.61, 3.71], pd = 99.6%, ps = 99.4%, BF_10_ = 8.14), with an inversion of this relationship during exercise (interaction effect, *β* = −0.93, 95% CI [−3.03, 1.13], pd = 81.3%, ps = 78.6%, BF_10_ = 0.52).

For composite indices, a similar pattern was observed, albeit only for the stress index. At rest, a negative association between the stress index and absolute lymphocyte counts was observed, with an 80% posterior probability (*β* = −0.56, 95% CI [−1.85, 0.75], pd = 80%, ps = 75.5%, BF_10_ = 0.32). Additionally, a 77.7% posterior probability of an interaction effect suggested an inversion of this initial relationship during exercise (interaction effect, *β* = 0.7, 95% CI [−1.16, 2.45], pd = 77.7%, ps = 74.4%, BF_10_ = 0.41). This provides compelling evidence that baseline immune cells interact with cardiac autonomic control and modify its trajectories during exercise‐induced strain.

#### 3.3.2 B Cells

For B‐cell counts, we observed a 93% posterior probability of a discrete positive association with resting‐state RMSSD (*β* = 0.35, 95% CI [−0.11, 0.82], pd = 93%, ps = 85.7%, BF_10_ = 0.24), but no other time‐domain parameters at rest or during exercise.

For frequency parameters, we observed a 98.9% posterior probability of a positive association with resting‐state HF (*β* = 0.58, 95% CI [0.07, 1.07], pd = 98.9%, ps = 97%, BF_10_ = 1.08), and a 98.6% posterior probability of an inversion of this relationship for during‐exercise measurements of HF (interaction effect, *β* = −0.64, 95% CI [−1.21, −0.09], pd = 98.6%, ps = 97%, BF_10_ = 1.16). Similar effects were observed with LF and VLF in resting conditions, where a 95.3% posterior probability and a 99.4% posterior probability of a negative effect were observed for absolute B‐cell counts (effect on LF, *β* = −0.43, 95% CI [−0.94, 0.07], pd = 95.3%, ps = 90.2%, BF_10_ = 0.37; effect on VLF, *β* = −0.7, 95% CI [−1.22, −0.17], pd = 99.4%, ps = 98.7%, BF_10_ = 2.26). However, a 90.4% and a 99.3% posterior probability of an interaction effect suggested that the during‐exercise LF and VLF relationship with absolute B‐cell counts inverted from the resting conditions (interaction effect for LF, *β* = 0.44, 95% CI [−0.25, 1.11], pd = 90.4%, ps = 84.1%, BF_10_ = 0.27; interaction effect for VLF, *β* = 0.88, 95% CI [0.18, 1.57], pd = 99.3%, ps = 98.7%, BF_10_ = 2.7).

Composite indices displayed more subtle relationships. Even though we observed an absence of effect of B‐cell counts on baseline PNS (*β* = −0.08, 95% CI [−0.68, 0.52], pd = 60.5%, ps = 47.8%, BF_10_ = 0.1), SNS (*β* = −0.05, 95% CI [−0.46, 0.32], pd = 60.8%, ps = 40.9%, BF_10_ = 0.07), and the Stress index (*β* = 0.02, 95% CI [−0.44, 0.44], pd = 52.7%, ps = 35.2%, BF_10_ = 0.07), the effects varied with during‐exercise measurements. During exercise, greater B‐cell counts were associated with a 78.4% posterior probability of reduced PNS (interaction effect, *β* = −0.33, 95% CI [−1.16, 0.48], pd = 78.4%, ps = 70.4%, BF_10_ = 0.18), an 89.9% posterior probability of reduced SNS (interaction effect, *β* = −0.33, 95% CI [−0.83, 0.18], pd = 89.9%, ps = 81.5%, BF_10_ = 0.19), and a 96.7% posterior probability of reduced Stress index (interaction effect, *β* = −0.56, 95% CI [−1.16, 0.04], pd = 96.7%, ps = 93.7%, BF_10_ = 0.57).

#### 3.3.3 T Cells

Absolute T‐cell counts showed a positive association with resting mean RR (*β* = 1.16, 95% CI [−0.27, 2.51], pd = 94.6%, ps = 93%, BF_10_ = 0.88), but a negative interaction effect during exercise (interaction effect, *β* = −0.77, 95% CI [−2.09, 0.41], pd = 89.3%, ps = 86.1%, BF_10_ = 0.45).

For absolute T‐cell counts, we observed an 83% and 99.1% posterior probability of a negative association with baseline LF (*β* = −0.71, 95% CI [−2.15, 0.77], pd = 83%, ps = 79.1%, BF_10_ = 0.39), and VLF levels (β = −1.82, 95% CI [−3.3, −0.26], pd = 99.1%, ps = 98.7%, BF_10_ = 3.73). Additionally, a 76.7% and 85.8% posterior probability of an inversion of this effect was observed during exercise measurements (interaction effect for LF, *β* = 0.73, 95% CI [−1.4, 2.56], pd = 76.7%, ps = 73.6%, BF_10_ = 0.45; interaction effect for VLF, *β* = 1.11, 95% CI [−0.95, 3.15], pd = 85.8%, ps = 83.3%, BF_10_ = 0.61). No other associations were observed between HRV measurements and absolute T‐cell counts.

#### 3.3.4 CD4+ and CD8+ T Cells

For CD4+ and CD8+ cell counts, no individual associations were observed with autonomic parameters. However, the CD4+/CD8+ ratio exhibited a 99.8% posterior probability of a negative association with resting RMSSD (*β* = −0.58, 95% CI [−0.96, −0.2], pd = 99.8%, ps = 99.4%, BF_10_ = 4.89) and a 98.6% posterior probability of a negative association with resting SDNN (*β* = −0.41, 95% CI [−0.78, −0.05], pd = 98.6%, ps = 95%, BF_10_ = 0.69). Additionally, we observed a 99.5% and 99.7% posterior probability of an inversion of the observed effect during exercise measurements for RMSSD (interaction effect, *β* = 0.56, 95% CI [0.14, 1], pd = 99.5%, ps = 98.2%, BF_10_ = 1.69) and SDNN (interaction effect, *β* = 0.64, 95% CI [0.2, 1.1], pd = 99.7%, ps = 99.1%, BF_10_ = 3.57).

For frequency domains, we observed a 98.9% posterior probability of similar behavior with resting HF (*β* = −0.47, 95% CI [−0.87, −0.07], pd = 98.9%, ps = 96.5%, BF_10_ = 0.85), and an 89.7% posterior probability of similar behavior with resting LF values (*β* = −0.27, 95% CI [−0.65, 0.17], pd = 89.7%, ps = 78.3%, BF_10_ = 0.16). Moreover, we observed a 99% and 99.7% posterior probability of an inversion of the relationship with the CD4+/CD8+ ratio during exercise measurements for HF (interaction effect, *β* = 0.53, 95% CI [0.09, 0.98], pd = 99%, ps = 97.2%, BF_10_ = 1.18) and LF values (interaction effect, *β* = 0.76, 95% CI [0.24, 1.33], pd = 99.7%, ps = 99.1%, BF_10_ = 3.39). Similarly, but with more uncertainty, a 72.8% posterior probability suggested reduced VLF at rest with an increasing CD4+/CD8+ ratio (*β* = −0.13, 95% CI [−0.57, 0.28], pd = 72.8%, ps = 55.7%, BF_10_ = 0.09). A 94.1% posterior probability of a similar interaction effect was observed for the during‐exercise response (interaction effect, *β* = 0.46, 95% CI [−0.12, 1.02], pd = 94.1%, ps = 89.2%, BF_10_ = 0.34).

#### 3.3.5 NK Cells

Finally, we observed varying cardiac autonomic responses related to NK cells and their subpopulations, with these effects being dependent on the abundance of cell subsets at baseline. The specific influence of NK cell quantities on HRV is domain‐specific.

For time‐domain parameters, greater baseline NK cell counts were associated with an 87.3% posterior probability of increased mean RR at rest (*β* = 0.37, 95% CI [−0.29, 1.03], pd = 87.3%, ps = 79.9%, BF_10_ = 0.22), but a 91.1% posterior probability of reduced mean RR during exercise (interaction effect, *β* = −0.39, 95% CI [−0.97, 0.16], pd = 91.1%, ps = 84.2%, BF_10_ = 0.23).

In the spectral components of HRV, absolute NK cell counts displayed an inverse relationship with baseline VLF (*β* = −0.69, 95% CI [−1.41, 0.07], pd = 96.5%, ps = 94.1%, BF_10_ = 0.66), with no other effects observed in this domain.

Composite indices, on the other hand, revealed dynamics not observed at rest. NK cell counts were not associated with different PNS levels at rest (*β* = −0.03, 95% CI [−0.84, 0.82], pd = 52.8%, ps = 43.1%, BF_10_ = 0.14), but they were linked to an 86.7% posterior probability of decreased during‐exercise PNS levels (*β* = 0.65, 95% CI [−0.56, 1.77], pd = 86.7%, ps = 82.9%, BF_10_ = 0.37). Additionally, NK cell counts were proportionally associated with an 84.1% posterior probability of increased Stress index at rest (*β* = 0.31, 95% CI [−0.3, 0.92], pd = 84.1%, ps = 75.5%, BF_10_ = 0.18).

#### 3.3.6 CD56 ^bright^ and CD56 ^dim^ NK Cells

When assessing NK subpopulations, the effects observed on exercise‐induced cardiac autonomic response become clearer. For instance, greater CD56‐dim NK cell counts were associated with a 98.1% posterior probability of increased mean RR (*β* = 0.42, 95% CI [0.02, 0.81], pd = 98.1%, ps = 94.5%, BF_10_ = 0.56). Conversely, this effect was reversed, with an 85.2% posterior probability that greater CD56‐bright NK cell counts were associated with reduced mean RR at rest (*β* = −0.24, 95% CI [−0.67, 0.21], pd = 85.2%, ps = 72.5%, BF_10_ = 0.13).

In the frequency domain, we observed a lack of influence from NK subpopulations on resting‐state HRV parameters. However, greater CD56‐dim NK cell counts were associated with a decrease in during‐exercise VLF measurements (interaction effect, *β* = −0.55, 95% CI [−1.17, 0.1], pd = 95.6%, ps = 92%, BF_10_ = 0.49). Conversely, CD56‐bright NK cell counts did not exert any effects on during‐exercise VLF relative to resting values (interaction effect, *β* = 0.02, 95% CI [−0.71, 0.77], pd = 51.8%, ps = 41.6%, BF_10_ = 0.12).

Composite domains exhibited properties similar to spectral components. The PNS index did not fluctuate with varying levels of either CD56‐bright (*β* = −0.02, 95% CI [−0.64, 0.6], pd = 52.5%, ps = 39.7%, BF_10_ = 0.11) or CD56‐dim (*β* = −0.02, 95% CI [−0.56, 0.5], pd = 53.3%, ps = 39%, BF_10_ = 0.09) NK cells at rest. However, during exercise, they displayed opposite effects on PNS. On one side, we observed that increasing levels of CD56‐bright NK cells were associated with an 89.9% posterior probability of decreased during‐exercise PNS levels (*β* = −0.55, 95% CI [−1.41, 0.3], pd = 89.9%, ps = 85%, BF_10_ = 0.32). Conversely, higher CD56‐dim NK cell counts were linked to a 95.5% posterior probability of an increase in during‐exercise PNS index (*β* = 0.64, 95% CI [−0.11, 1.35], pd = 95.5%, ps = 92.6%, BF_10_ = 0.55).

Similarly, NK subpopulations were not associated with varying resting SNS levels (effect of CD56‐bright, *β* = 0.02, 95% CI [−0.37, 0.42], pd = 53.9%, ps = 34.5%, BF_10_ = 0.07; effect of CD56‐dim, *β* = 0.09, 95% CI [−0.23, 0.45], pd = 71.1%, ps = 48.8%, BF_10_ = 0.07). However, a discrete 81.1% posterior probability of an increase in the Stress index at rest was observed, but only for increasing levels of CD56‐dim NK cells (*β* = 0.17, 95% CI [−0.21, 0.56], pd = 81.1%, ps = 64.6%, BF_10_ = 0.1). However, similar to what we previously observed with the other HRV parameter domains, an inversion of the effect of immune profile on HRV occurred during exercise. More precisely, an increase in CD56‐dim NK cells was associated with a 92.4% posterior probability of an increase in SNS (*β* = 0.32, 95% CI [−0.12, 0.78], pd = 92.4%, ps = 83.6%, BF_10_ = 0.2) and a 95.5% posterior probability of an increase in the Stress index during exercise‐related strain (*β* = 0.45, 95% CI [−0.08, 0.97], pd = 95.5%, ps = 90.7%, BF_10_ = 0.36), respectively.

**FIGURE 3:**
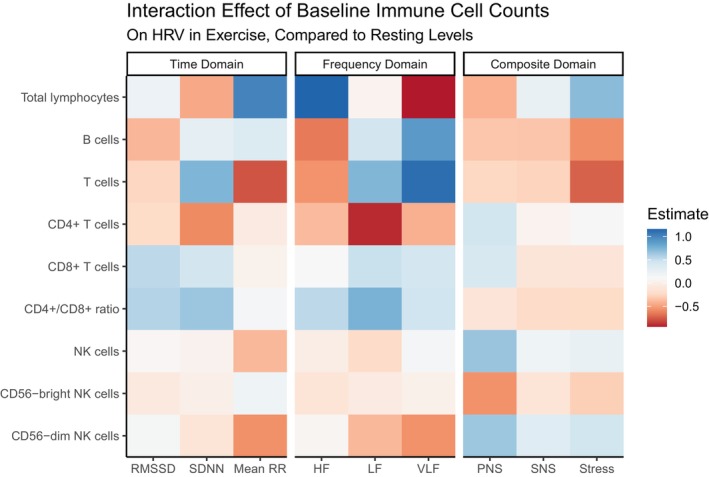
Interaction effects from autonomic‐immune models. This figure illustrates how an increase of one standard deviation unit in a specific immune cell population (rows) influences the exercise‐induced change in each HRV domain (columns), relative to resting values. More intense blue indicates that an increase in the specific immune cell is associated with a more marked increase in the corresponding HRV parameter during exercise, compared to rest. Conversely, more intense red indicates that an increase in the specific immune cell is associated with a more marked decrease in the HRV parameter during exercise, compared to rest. All depicted effects are adjusted for confounders.

Interaction effects illustrating the exercise‐induced dynamics across different HRV domains, as influenced by standardized cell counts for each immune phenotype, are depicted in Figure 3.

In an exploratory PBMC subsample (*n* = 9), we confirmed the presence of CD3+CD4+CD25^hiCD127^low Tregs and several B‐cell subsets, including naive, unswitched memory, IgG‐switched memory, CD21^low^ B cells and CD19+CD24^hiCD38^hi regulatory/transitional B cells. Median frequencies were ~3% Tregs among CD4+ T cells, ~61% naive B cells, ~32% unswitched and ~5% switched memory B cells, ~2% CD21^low^ B cells and ~11% Bregs among CD19+ B cells (Table S3 and Figure S2).

In the PBMC subsample (*n* = 9), exploratory descriptive stratification by sex did not reveal systematic differences across Tregs, naïve and memory B cells, CD21^low^ B cells, IgM+ Bregs, or CD4+CD8+ T cells (Figure S4), and no sex‐stratified models were fitted due to the small sample size.

The effect of confounding variables can be observed in the section “Secondary analysis” of Appendix S1.

## 4 Discussion

This study investigated the dynamic interplay between baseline immune cell profiles and cardiac autonomic trajectories during rest, exercise, and recovery in older adults. Our findings extend earlier data showing that acute exercise elicits significant, time‐dependent changes in cardiac autonomic control, characterized by a shift towards sympathetic dominance during exercise and a gradual return towards baseline parasympathetic tone during recovery. In addition, to our knowledge this study is one of the first in older adults to show that the baseline composition of specific immune cell subsets are associated with different temporal patterns and magnitudes of these exercise‐induced autonomic trajectories, suggesting that the immune system's basal state actively shapes the cardiac autonomic response to acute physiological stress in aging.

### 4.1 Autonomic Responses to Exercise in Older Adults

As anticipated, exercise elicited significant and time‐dependent dynamics in HRV parameters, consistent with a robust autonomic response to metabolic demands and physiological stress (Castillo‐Aguilar et al. 2023, 2025; Castro et al. 2024). Reductions in time‐ and frequency‐domain indices (e.g., RMSSD and HF) during exercise reflect expected parasympathetic withdrawal and/or sympathetic increase necessary to elevate heart rate and adapt to transient physical strain. Partial recovery post‐exercise is closely related to the high heterogeneity in older adults' autonomic recovery capacity, potentially influenced by factors beyond exercise intensity, such as fitness and health (Mosley and Laborde 2024; Reis et al. 2024), and, as our data suggest, baseline immune profile. Several cardiac autonomic indices, derived from HRV parameters, mirrored these shifts. We observed that many of the domain‐specific changes in exercise‐induced cardiac autonomic dynamics depended on specific immune cell populations, inducing domain‐specific responses, reflecting the interlinked dependence between physiological strain imposed by exercise and the baseline neuroimmune state.

### 4.2 Immune Profile Shapes Autonomic Trajectories

Our most significant finding is that baseline immune cell counts are associated with the dynamic trajectory of autonomic changes before and during exercise, rather than merely being static correlates of resting HRV. Significant interaction terms between specific baseline immune subsets and the “time relative to exercise” variable indicate that individuals with different baseline immune profiles exhibited distinct patterns of autonomic response and recovery. This suggests that the pre‐existing state of the immune system, potentially reflecting aspects of immunosenescence and inflammaging, influences the capacity of the autonomic nervous system to adapt to and recover from acute physiological stress. This goes beyond simple correlation, indicating a more complex, context‐dependent modulation where the immune system's basal state influences the shape of the autonomic response curve during exercise‐induced stress.

### 4.3 Specific Immune Cell Associations With Autonomic Dynamics

Specifically, baseline total lymphocyte counts were inversely associated with resting HF (parasympathetic tone) and positively with resting VLF. More importantly, interaction effects revealed an inversion during exercise. Higher baseline total lymphocytes related to a less pronounced relative decrease in VLF and attenuated inverse resting HF relationship during exercise. This dynamic modulation points towards a complex, context‐dependent interaction, possibly driven by differential lymphocyte trafficking or altered immune‐mediated signaling during exercise stress (Wu et al. 2021; Udit et al. 2022).

Further detail came from specific lymphocyte subsets. Baseline B‐cell counts showed a positive association with resting RMSSD and HF, but interaction terms indicated higher baseline B‐cells linked to a greater relative HF reduction during exercise. This suggests that while higher baseline B‐cells might correlate with better resting tone, this profile is also associated with a more pronounced parasympathetic withdrawal or sympathetic surge during acute stress, potentially indicating a less resilient autonomic response. Similarly, baseline T‐cell counts and the CD4+/CD8+ ratio, markers linked to immunosenescence (Jin et al. 2024; Teodoro et al. 2025), were inversely associated with resting HRV parameters (VLF for T‐cells; RMSSD, SDNN, HF for CD4+/CD8+ ratio). Critically, interaction effects showed that higher baseline T‐cell and CD4+/CD8+ profiles were linked with an inversion or attenuation of these negative associations during exercise. In our cohort of community‐dwelling older adults, variation in the CD4:CD8 ratio was associated with several HRV parameters. However, these associations should not be interpreted as evidence of a fully developed immunosenescent T‐cell profile. First, our phenotyping did not distinguish naïve, central‐memory, effector‐memory, and terminally differentiated CD8+ subsets, which are critical to characterizing immunosenescence. Second, CD4:CD8 ratios in this sample were largely above 1, whereas an inverted ratio (< 1) has been proposed as a more robust marker of immunosenescence and the so‐called “immunological risk phenotype” (Aiello et al. 2019). Finally, baseline NK cell counts also displayed an inverse association with resting VLF; interactions indicated higher NK counts attenuated or reversed this negative relationship during and after exercise, suggesting a specific involvement of NK cells in VLF modulation during stress/recovery. Expanding on this, our analysis of NK subpopulations revealed even clearer patterns during exercise‐induced cardiac autonomic response.

Specifically, NK subpopulations exhibited distinct baseline roles in autonomic modulation roles. A higher abundance of CD56‐dim NK cells appeared linked to increased resting mean RR, suggesting enhanced parasympathetic influence, while CD56‐bright NK cells seemed associated with reduced mean RR. During exercise, our findings became even more telling. Increased CD56‐dim NK cells were associated with decreased VLF measurements. It has been suggested that a shift towards a higher proportion of CD56‐dim NK cells may be associated with a more adaptative autonomic response, possibly through cytokine up‐regulation and indirect inflammatory signaling mechanisms (Solana et al. 2014; Yu et al. 2018; Seymour et al. 2022). Critically, composite indices revealed opposite effects on PNS. Higher CD56‐bright NK cells were associated with decreased during‐exercise PNS, possibly reflecting less buffered parasympathetic withdrawal. In contrast, increased CD56‐dim NK cells were linked to increased during‐exercise PNS, suggesting they might actively sustain parasympathetic activity or temper sympathetic surge. Furthermore, the increased SNS and Stress index during exercise‐related strain, linked to elevated CD56‐dim NK cells, suggests a potential subset‐specific influence of NK cells on the acute physiological stress response, likely via localized neuro‐immune interactions or context‐dependent modulation of inflammatory mediators.

### 4.4 Potential Underlying Mechanisms

These findings collectively support the notion that the baseline immune landscape, shaped by age‐related changes such as immunosenescence and inflammaging, modulates the dynamic autonomic response to acute stress. While associational, several potential mechanisms could underlie this crosstalk.

Firstly, baseline immune cell profiles contribute to the prevailing cytokine environment. Inflammaging‐associated cells maintain elevated pro‐inflammatory cytokines (e.g., TNF‐alpha and IL‐6) at rest (Li et al. 2023; Bender et al. 2025), which can rapidly increase during exercise (Reis et al. 2024). Elevated baseline cytokines can acutely influence autonomic control by acting on central autonomic nuclei, modulating peripheral nerve activity (e.g., sensitizing vagal fibers), or altering cardiac sensitivity to neurotransmitters (Shi et al. 2011; Lamotte et al. 2021). Anti‐inflammatory profiles might support efficient recovery.

Moreover, immune cells express functional receptors for autonomic neurotransmitters (norepinephrine, acetylcholine) and stress hormones (cortisol) (Wu et al. 2021). With distinct patterns in different cell populations, for instance glucocorticoid receptors show moderate expression in T‐cells but higher expression in B‐cells and NK‐cells (Gotovac et al. 2003). Baseline immune state (activation or senescence) could alter the expression, density, or signaling efficiency of these receptors. This altered landscape might influence the systemic response to the surge in autonomic and hormonal activity during exercise (Reis et al. 2024), potentially affecting immune cell trafficking or mediator release in a way that feeds back to modify autonomic outflow or end‐organ responsiveness (Mueller 2022).

The autonomic–immune interface is likely mediated, at least in part, by circulating catecholamines. Acute sympathetic activation during exercise triggers the release of epinephrine, which acts as a powerful neurotransmitter–hormone on immune cells. Through β‐adrenergic signaling, epinephrine can rapidly mobilize CD8+ T cells and NK cells into and out of the circulation, contributing to the characteristic transient lymphocytosis during exercise and the subsequent redistribution of cytotoxic subsets during recovery (Kruger and Mooren 2007; Gleeson et al. 2011; Bigley and Simpson 2015). Our HRV findings, which reflect both vagal withdrawal and sympathetic drive, are therefore consistent with a scenario in which autonomic shifts are coupled to catecholamine‐dependent trafficking of effector lymphocytes.

Additionally, metabolically active immune cells interact closely with both systemic and local metabolic pathways (Mueller 2022). Exercise profoundly alters the metabolic environment (Dellacqua et al. 2024). The baseline metabolic state influenced by immune cells, particularly in the context of inflammaging which can induce metabolic dysregulation, could feed back to influence autonomic regulation centers or peripheral nerve function during exercise's heightened metabolic state (Meyer‐Lindemann et al. 2023). Immune‐derived metabolites or substrate consumption might impact neuronal energy status or signaling (Kovacs et al. 2025).

Although our primary analyses focused on major lymphocyte subsets in whole blood, we performed extended immunophenotyping in a small PBMC subsample to document regulatory populations. These exploratory data confirmed the presence of CD3+CD4+CD25^hiCD127^low Tregs and CD19+CD24^hiCD38^hi Bregs, together with naïve, unswitched and switched memory and CD21^low^ B‐cell subsets, at frequencies broadly consistent with previous reports in older adults (Blanco et al. 2018; Colucci et al. 2023). Across these subsets we observed modest inter‐individual variability but no systematic shifts by sex, in line with a relatively healthy, community‐dwelling cohort. Given the limited sample size (*n* = 9), these findings are presented descriptively and were not incorporated into the Bayesian regression models, but they provide useful biological context and a proof‐of‐principle for future, more deeply phenotyped studies linking specific regulatory B‐ and T‐cell compartments with autonomic responses to stress.

Finally, specific immune cells might interact directly with autonomic nerve endings in peripheral tissues, forming structures akin to “neuro‐immune synapses”. The number, activation state, or spatial distribution of immune cells at baseline can influence the release or uptake of neurotransmitters, such as norepinephrine or acetylcholine during periods of high autonomic activity, thereby directly modulating local autonomic control of the heart or vasculature (Khanmammadova et al. 2023).

### 4.5 Clinical and Physiological Implications

These findings have important clinical and physiological implications, particularly for aging populations. They offer a potential explanation for heterogeneity in physiological responses to exercise among older adults. Individuals with different underlying immune profiles may exhibit varying degrees of autonomic adaptability during stress, potentially affecting acute cardiovascular risk. Understanding this dynamic crosstalk could pave the way for more personalized approaches to exercise prescription and stress management in older adults. Baseline immune assessment, as described here, is readily available in the clinical setting and might help identify individuals who could benefit from tailored exercise regimens or even pre‐exercise interventions (e.g., nutritional, mild anti‐inflammatory approaches) based on their immune profile. This integrated view suggests that strategies aimed at mitigating immunosenescence or inflammaging might also enhance autonomic resilience and cardiovascular health in aging.

### 4.6 Strengths and Limitations

The present study has several strengths, including the focus on older adults, a population where both immune and autonomic dysfunction are prevalent and clinically significant (ps‐ and BF‐wise). We employed a detailed assessment of dynamic HRV changes across distinct exercise phases, moving beyond static resting measures to capture the crucial response to acute stress. Quantification of specific baseline immune cell subsets provides more granularity than total leukocyte counts. A robust Bayesian modeling approach allowed comprehensive analysis of complex interactions while controlling for relevant confounders, providing probabilistic interpretations. Adherence to the SEXIT framework ensured transparent reporting (Makowski et al. 2019).

However, several limitations must be acknowledged. The study design is associational, precluding definitive causal conclusions. Future functional experiments are required. We measured baseline immune profiles statically; acute exercise also induces dynamic immune changes influencing autonomic control (Meyer‐Lindemann et al. 2023; Dellacqua et al. 2024). Concurrent measurement of dynamic immune parameters alongside HRV during/after exercise would provide a more complete picture of bidirectional crosstalk. Findings may vary depending on the type, intensity, or duration of exercise (Rocha‐Santos et al. 2020). Unmeasured factors could still influence associations. Immunogenetic assessment of the full immune repertoire could further help in understanding the underlying mechanisms (Koning et al. 2019). A further limitation is the absence of systematic data on menstrual status and use of estrogen therapy in female participants. Although all models were adjusted for sex, age and individual characteristics (including physical‐performance measures), unmeasured variability in hormonal status may have contributed to residual confounding in the estimated effects. Finally, we lacked direct measures of cytokine levels, receptor expression, or signaling pathways needed to elucidate functional changes.

### 4.7 Future Research Directions

These limitations highlight crucial directions for future research. Longitudinal studies on immune and autonomic plasticity, incorporating interventions, are needed. Investigations incorporating concurrent, dynamic measurements of immune mediators and detailed autonomic indices during acute stress are invaluable. Mechanistic studies using in vitro or animal models could explore specific pathways. Translational research should explore if targeting immune pathways (e.g., via lifestyle) can improve autonomic resilience and exercise capacity in older adults. Large‐scale studies are needed to assess the predictive value of combining baseline immune markers and dynamic autonomic responses for long‐term health outcomes in the aging population.

## 5 Conclusion

Our research emphasizes that an individual's baseline immune profile plays a crucial role in shaping how the heart's autonomic system responds to exercise in older adults. These findings underscore the importance of considering the interplay between the immune and autonomic systems when assessing physiological resilience during aging. By moving beyond traditional, static measurements and focusing on how these two essential systems interact dynamically under stress, we gain deeper insight into the aging process. This approach not only enhances our understanding of how aging affects the body but also opens new avenues for research and intervention aimed at helping older adults maintain their health and functionality for longer.

## Author Contributions

Conceptualization, M.C.‐A.; Data curation, M.C.‐A.; Investigation, M.C.‐A., C.N.‐E.; Methodology, M.C.‐A., C.N.‐E., L.S.V.; Supervision, C.N.‐E.; Formal analysis, M.C.‐A.; Visualization, M.C.‐A.; Writing – original draft, M.C.‐A., C.N.‐E.; Writing – review and editing, M.C.‐A., L.S.V., C.P., R.U.‐P., M.A.N., C.N.‐E. All authors have read and agreed to the published version of the manuscript.

## Funding

This work was funded by the Chilean National Agency for Research and Development (ANID) under project Fondecyt Regular N°1250474.

## Ethics Statement

Ethical approval was obtained from the Ethics Committee of the University of Chile (ACTA No. 029 − 18/05/2022) and the Ethics Committee of the University of Magallanes (No. 008/SH/2022).

## Consent

All participants received detailed information regarding the study objectives, procedures, and potential implications. Informed consent was obtained to ensure ethical compliance and participant autonomy.

## Conflicts of Interest

The authors declare no conflicts of interest.

## Supporting information


**Appendix S1:** acel70428‐sup‐0001‐AppendixS1.docx.

## Data Availability

The data that support the findings of this study are available on request from the corresponding author. The data are not publicly available due to privacy or ethical restrictions.
