# Supplementary material for: The Immune‐Autonomic Interface in Aging: Baseline Immune Profile Shapes Cardiac Autonomic Response to Exercise
Source: Aging Cell. 2026 Mar 6;25(3):e70428. doi: 10.1111/acel.70428 (PMC12965845; doi:10.1111/acel.70428)
Supplement: Supplementary file 1 — Appendix S1: acel70428‐sup‐0001‐AppendixS1.docx. [file ACEL-25-e70428-s001.docx]

**Title**: The Immune-Autonomic Interface in Aging: Baseline Immune Profile Shapes Cardiac Autonomic Response to Exercise

**Running Title**: The Immune-Autonomic Interface in Aging

**Authors**: Matías Castillo-Aguilar^1,2^, Lindybeth Sarmiento Varón^1,2^, Carolina Pérez^1^, Roberto Uribe-Paredes^3,4^, Marcelo A. Navarrete^1,2^*, Cristian Nuñez-Espinosa^1,2^*

^1^ Centro Asistencial Docente e Investigación (CADI), Universidad de Magallanes, Punta Arenas, Chile.

^2^ Escuela de Medicina, Universidad de Magallanes, Punta Arenas, Chile.

^3^ Departamento de Ingeniería en Computación, Universidad de Magallanes, Punta Arenas, Chile.

^4^ Centre for Biotechnology and Bioengineering, CeBiB, Universidad de Chile, Santiago, Chile.

### *Correspondence

Cristian Núñez-Espinosa y Marcelo Navarrete Signorile, Escuela de Medicina, Universidad de Magallanes, Punta Arenas, Chile. Centro Asistencial de Docencia e Investigación CADI-UMAG, Chile. e-mail: [cristian.nunez@umag.cl](mailto:cristian.nunez@umag.cl). Address: Avenida Bulnes 01855, Box 113-D. Phone: +56 61 2201411.

# Material and methods

## Gating strategy

Data analysis followed a hierarchical gating strategy. Viable cells were first identified on a forward-scatter versus side-scatter dot plot to establish the total-cell gate.

Lymphocytes were then gated as CD45 high with low side scatter. Within this lymphocyte gate, B cells were discriminated by CD19 expression in the absence of CD3, and T cells by CD3 expression without CD19. Further subdivision of T cells distinguished CD4+ helper T cells by co-expression of CD3 and CD4, and CD8+ cytotoxic T cells by co-expression of CD3 and CD8. Natural killer cells were identified as CD56+ in the absence of CD3.

Initial gating and region definitions were performed in the cytometer’s CytoExpert software using internal controls and expected population distributions; the resulting FCS files were then exported to FlowJo (Tree Star, Ashland, OR, USA) for final refinement and detailed characterization of the lymphocyte subpopulations.

Overall Gating strategy for major lymphocyte and NK-cell subsets in whole blood can be seen in figure S1, gating strategy for Tregs, memory B cells, CD21^low B cells and IgM+ Bregs in PBMCs figure S2.

**Figure S1**. Gating strategy for major lymphocyte and NK-cell subsets in whole blood from older adults. (A) Representative sequential gating on fresh whole blood: singlets (FSC-H vs FSC-A), “cells” (FSC-A vs SSC-A) and CD45+ lymphocytes (SSC-A vs CD45). (B) Within CD45+ lymphocytes, CD3+ T cells were selected and subdivided on a CD4 vs CD8 bivariate plot into CD4+CD8- helper T cells, CD4-CD8+ cytotoxic T cells, CD4+CD8+ double-positive T cells and CD4-CD8- double-negative/other T cells. (C) NK-cell panel. CD3 vs CD56 dot plot showing CD3+CD56- T cells, CD3+CD56+ NKT cells, CD3-CD56+ NK cells and CD3-CD56- double-negative cells. Within the CD3-CD56+ NK gate, CD56 expression was displayed on a one-dimensional histogram to define CD56^bright and CD56^dim NK subsets; CD3+CD56- T cells from the same sample served as an internal CD56-negative control to set the CD56+ threshold. All plots are gated on singlets and viable cells and are representative of fresh samples from the main cohort.

**Figure S2**. Gating strategy for Tregs, memory B cells, CD21^low B cells and IgM+ Bregs in PBMCs from older adults. (A) Representative sequential gating on whole blood/PBMCs: singlets (FSC-H vs FSC-A), intact cells, viable 7-AAD- cells and CD45+ lymphocytes (SSC-A vs CD45).(B) Treg panel. Within CD45+ lymphocytes, CD3+CD4+ T cells were selected and regulatory T cells (Tregs) identified as CD25hiCD127low/- cells. Frequencies are expressed as a percentage of CD3+CD4+ T cells. (C) Memory B-cell panel. CD3-CD19+ B cells were gated from CD45+ lymphocytes and subdivided on IgG vs CD27 into naïve (IgG-CD27-), unswitched memory (IgG-CD27+) and switched memory (IgG+CD27+) B cells. CD21low B cells were defined within CD19+ cells using a CD21 histogram and backgating on a CD27 vs IgG dot plot, Red dots: CD21^low B cells; grey/blue cloud: all CD19+ B cells. (D) Breg panel. CD3-CD19+IgM+ B cells were analysed on CD24 vs CD38 to identify CD24hiCD38hi regulatory/transitional B cells (Bregs), alongside mature naïve, memory and plasmablast/plasma cell compartments. All plots are gated on singlets and viable cells and are representative of the PBMC subsample (n = 9).

Additionally, predicted exercise-induced cardiac autonomic modulation by standardized CD3+CD8+CD4+ and CD3+CD8-CD4- immune cell counts can be observed in figure S3, and the exploratory distribution of Tregs, naïve and memory B cells, CD21^low B cells, IgM+ Bregs and CD4+CD8+ T cells in the PBMC subsample can be observed in Figure S4.

**Figure S3**. Predicted exercise-induced cardiac autonomic modulation by standardized CD3+CD8+CD4+ and CD3+CD8-CD4- immune cell counts across discrete time frames. The trajectories illustrate the relative dynamics of HRV parameters in response to exercise, based on immune cell count groups standardized as z-scores. ‘Pre’ represents the baseline resting condition, ‘Peri’ represents the in-exercise HRV measurements, and ‘Post’ represents the post-exercise 5-minute recovery window. All depicted effects are adjusted for confounders.

**Figure S4**. Exploratory distribution of Tregs, naïve and memory B cells, CD21^low B cells, IgM+ Bregs and CD4+CD8+ T cells in the PBMC subsample (n = 9), stratified by sex, CD4:CD8 ratios and NK cells. Boxes represent mean and bootstrapped 95% confidence interval.

**Table S1**. Subsample characteristics of Tregs, naïve and memory B cells, CD21^low B cells, IgM+ Bregs and CD4+CD8+ T cells in the PBMC subsample (n = 9), stratified by sex, CD4:CD8 ratios and NK cells. Values are presented as mean ± SD and median (IQR), expressed as percentage of CD4+ or CD19+ cells as indicated.

| Subpopulation | Definition | Mean ± SD | Median (IQR) |
| --- | --- | --- | --- |
| Tregs | CD3+CD4+CD25^hi CD127^low/ CD4+ | 2.88 ± 0.69 | 3.19 (2.39–3.33) |
| Naïve B | CD19+CD27- / CD19+ | 60.88 ± 8.93 | 62.9 (52.4–65.6) |
| Unswitched memory B | CD19+CD27+IgG- / CD19+ | 32.40 ± 7.53 | 31.6 (27.2–38.6) |
| Switched memory B | CD19+CD27+IgG+ / CD19+ | 4.70 ± 2.42 | 4.64 (2.27–6.33) |
| CD21^low B | CD19+CD21^low / CD19+ | 1.86 ± 0.55 | 2.05 (1.38–2.27) |
| Bregs | CD19+CD24^hiCD38^hi / CD19+ | 11.25 ± 4.16 | 10.8 (8.4–12.3) |

## Statistical analysis

To evaluate the effect of exercise on HRV, we built two families of generalized linear mixed-effects models with subject-level random intercepts. The first family quantified how exercise modified HRV metrics in the time domain (RMSSD, SDNN, mean R-R interval), the frequency domain (HF, LF, VLF power), and composite indices (PNS, SNS, and Stress index). The second family extended these models by incorporating the absolute counts of distinct immune cell phenotypes and their interaction with the timing of HRV measurement. Both model families controlled for the marginal effects of sex, age, systolic, diastolic, mean and pulse arterial pressures, body mass index, body fat percentage, lean muscle mass, steps performed in the TMST, time to complete the TUG, and repetitions in the CSTS test, thereby isolating the unique contributions of exercise and immune parameters. Model fitting employed the No-U-Turn Sampler (a variant of Hamiltonian Monte Carlo) as implemented in the brms (v2.22.0) and rstan (v2.32.6) packages in R (R Core Team 2021), using four chains with 2,000 warm-up iterations and 3,500 sampling iterations each, yielding 14,000 post-warm-up samples per parameter.

Fixed-effect coefficients were assigned weakly regularizing normal priors centered at zero with a standard deviation of three ($\beta\mathcal{\sim N}\left( 0,3 \right)$), constraining extreme estimates and enhancing sampler stability. The standard deviation of the subject-level random intercepts was given a truncated Student-t prior with three degrees of freedom, location parameter one, scale parameter three, and a lower bound of zero ($\sigma_{\text{ID}}\sim\text{Student-t}\left( \nu=3,\mu=1,\sigma=3 \right)$, $\sigma_{\text{ID}}\geq0$), which permits realistic interindividual variability while limiting undue spread from outliers and facilitating efficient exploration of the posterior space (Van de Schoot et al. 2021).

Sampling convergence and stability were assessed by verifying that the potential scale reduction factor ($\hat{R}$) were less than 1.01 and that the effective sample were at least 1,000 for every parameter. We also inspected trace plots visually and performed posterior predictive checks to confirm appropriate chain mixing and that model predictions aligned with observed data distributions.

# Model Estimates

In the following sections, the complete model estimates, in addition to convergence metrics, are presented without immune cell adjustment and with immune cell adjustment. All models are adjusted for confounding factors, such a body composition, functional performance and basic sociodemographic factors, such as age and sex.

## Without immune cell influence

**Table S2**. Model estimates and 95% credible interval based on highest density interval (HDI) from the posterior distribution on time-domain parameters from the HRV components. These model effects are adjusted for confounders but not for baseline immune profile. pd, probability of direction; ps, probability of significance; BF, Bayes Factor in favor of the alternative hypothesis; ESS, effective sample size; R-hat, Potential reduction scale factor.

| Variable | Standardized Effect | 95% HDI | pd | ps | BF | ESS | R-hat |
| --- | --- | --- | --- | --- | --- | --- | --- |
| RMSSD \| Intercept | -0.03 | [-0.46, 0.4] | 0.555 | 0.377 | 0.07 | 8471.4 | 1.001 |
| SDNN \| Intercept | -0.23 | [-0.63, 0.18] | 0.865 | 0.734 | 0.13 | 10076.9 | 1.000 |
| Mean R-R \| Intercept | 0.73 | [0.29, 1.17] | 0.999 | 0.997 | 16.43 | 6934.4 | 1.000 |
| RMSSD \| Time-to-exercise [Peri] | -1.10 | [-1.33, -0.88] | 1.000 | 1.000 | 6213.68 | 11284.4 | 1.000 |
| RMSSD \| Time-to-exercise [Post] | 0.00 | [-0.23, 0.22] | 0.488 | 0.186 | 0.04 | 11215.3 | 1.000 |
| RMSSD \| Sex [Female] | 0.63 | [0.06, 1.24] | 0.980 | 0.960 | 0.84 | 8287.1 | 1.000 |
| RMSSD \| Age [+1 SD] | 0.04 | [-0.12, 0.19] | 0.677 | 0.212 | 0.03 | 9012.8 | 1.000 |
| RMSSD \| Systolic BP [+1 SD] | 0.03 | [-0.13, 0.19] | 0.644 | 0.189 | 0.03 | 8919.7 | 1.000 |
| RMSSD \| Diastolic BP [+1 SD] | 0.01 | [-0.18, 0.22] | 0.544 | 0.186 | 0.03 | 7516.6 | 1.000 |
| RMSSD \| BMI [+1 SD] | 0.36 | [0.03, 0.68] | 0.983 | 0.940 | 0.58 | 6659.5 | 1.000 |
| RMSSD \| Total fat [+1 SD] | -0.56 | [-0.98, -0.14] | 0.996 | 0.985 | 2.39 | 6319.2 | 1.000 |
| RMSSD \| Total muscle [+1 SD] | -0.25 | [-0.55, 0.06] | 0.945 | 0.828 | 0.17 | 7546.5 | 1.000 |
| RMSSD \| (SFT) TMST [+1 SD] | -0.15 | [-0.33, 0.05] | 0.934 | 0.681 | 0.10 | 8936.8 | 1.000 |
| RMSSD \| (SFT) TUG [+1 SD] | -0.11 | [-0.3, 0.06] | 0.899 | 0.556 | 0.07 | 8681.0 | 1.000 |
| RMSSD \| (SFT) CSTS [+1 SD] | 0.05 | [-0.12, 0.22] | 0.715 | 0.289 | 0.03 | 9507.5 | 1.000 |
| SDNN \| Time-to-exercise [Peri] | -0.98 | [-1.22, -0.75] | 1.000 | 1.000 | 6403.31 | 12357.0 | 1.000 |
| SDNN \| Time-to-exercise [Post] | 0.27 | [0.04, 0.51] | 0.987 | 0.918 | 0.48 | 11942.2 | 1.000 |
| SDNN \| Sex [Female] | 0.73 | [0.19, 1.25] | 0.995 | 0.989 | 2.89 | 9627.8 | 1.000 |
| SDNN \| Age [+1 SD] | 0.12 | [-0.02, 0.27] | 0.952 | 0.616 | 0.10 | 9972.7 | 1.000 |
| SDNN \| Systolic BP [+1 SD] | 0.00 | [-0.14, 0.14] | 0.507 | 0.091 | 0.02 | 11127.0 | 1.000 |
| SDNN \| Diastolic BP [+1 SD] | 0.13 | [-0.05, 0.31] | 0.918 | 0.621 | 0.09 | 8401.1 | 1.000 |
| SDNN \| BMI [+1 SD] | 0.33 | [0.03, 0.63] | 0.986 | 0.934 | 0.48 | 7753.3 | 1.000 |
| SDNN \| Total fat [+1 SD] | -0.55 | [-0.94, -0.18] | 0.997 | 0.989 | 2.52 | 7099.2 | 1.000 |
| SDNN \| Total muscle [+1 SD] | -0.20 | [-0.48, 0.08] | 0.921 | 0.764 | 0.13 | 8613.9 | 1.000 |
| SDNN \| (SFT) TMST [+1 SD] | -0.12 | [-0.29, 0.05] | 0.917 | 0.608 | 0.08 | 10713.5 | 1.000 |
| SDNN \| (SFT) TUG [+1 SD] | -0.23 | [-0.4, -0.07] | 0.997 | 0.942 | 1.32 | 9325.2 | 1.000 |
| SDNN \| (SFT) CSTS [+1 SD] | -0.03 | [-0.19, 0.13] | 0.636 | 0.178 | 0.03 | 10310.3 | 1.000 |
| Mean R-R \| Time-to-exercise [Peri] | -1.62 | [-1.78, -1.45] | 1.000 | 1.000 | 4388.73 | 16147.5 | 1.000 |
| Mean R-R \| Time-to-exercise [Post] | -0.40 | [-0.56, -0.24] | 1.000 | 1.000 | 604.38 | 17306.3 | 1.000 |
| Mean R-R \| Sex [Female] | -0.12 | [-0.73, 0.49] | 0.650 | 0.528 | 0.11 | 6761.0 | 1.000 |
| Mean R-R \| Age [+1 SD] | 0.07 | [-0.09, 0.24] | 0.819 | 0.379 | 0.04 | 6140.4 | 1.000 |
| Mean R-R \| Systolic BP [+1 SD] | 0.09 | [-0.07, 0.25] | 0.867 | 0.442 | 0.05 | 6429.5 | 1.000 |
| Mean R-R \| Diastolic BP [+1 SD] | -0.16 | [-0.37, 0.04] | 0.937 | 0.712 | 0.11 | 5811.7 | 1.000 |
| Mean R-R \| BMI [+1 SD] | -0.10 | [-0.43, 0.24] | 0.729 | 0.509 | 0.07 | 5436.8 | 1.000 |
| Mean R-R \| Total fat [+1 SD] | 0.13 | [-0.3, 0.54] | 0.726 | 0.558 | 0.09 | 5153.8 | 1.000 |
| Mean R-R \| Total muscle [+1 SD] | 0.09 | [-0.21, 0.42] | 0.720 | 0.477 | 0.06 | 6025.5 | 1.000 |
| Mean R-R \| (SFT) TMST [+1 SD] | -0.04 | [-0.23, 0.15] | 0.642 | 0.257 | 0.04 | 6578.1 | 1.000 |
| Mean R-R \| (SFT) TUG [+1 SD] | -0.01 | [-0.19, 0.17] | 0.532 | 0.153 | 0.03 | 6990.9 | 1.001 |
| Mean R-R \| (SFT) CSTS [+1 SD] | -0.10 | [-0.28, 0.07] | 0.869 | 0.497 | 0.05 | 6926.3 | 1.000 |

**Table S3**. Model estimates and 95% credible interval based on highest density interval (HDI) from the posterior distribution on frequency-domain parameters from the HRV components. These model effects are adjusted for confounders but not for baseline immune profile. pd, probability of direction; ps, probability of significance; BF, Bayes Factor in favor of the alternative hypothesis; ESS, effective sample size; R-hat, Potential reduction scale factor.

| Variable | Standardized Effect | 95% HDI | pd | ps | BF | ESS | R-hat |
| --- | --- | --- | --- | --- | --- | --- | --- |
| HF \| Intercept | -0.02 | [-0.51, 0.48] | 0.533 | 0.375 | 0.08 | 5933.3 | 1.000 |
| LF \| Intercept | -0.29 | [-0.72, 0.16] | 0.900 | 0.801 | 0.17 | 7603.3 | 1.000 |
| VLF \| Intercept | -0.31 | [-0.76, 0.14] | 0.912 | 0.821 | 0.19 | 8553.2 | 1.000 |
| HF \| Time-to-exercise [Peri] | -1.02 | [-1.25, -0.79] | 1.000 | 1.000 | 6340.75 | 13236.7 | 1.000 |
| HF \| Time-to-exercise [Post] | 0.07 | [-0.17, 0.3] | 0.711 | 0.390 | 0.05 | 12600.8 | 1.000 |
| HF \| Sex [Female] | 0.54 | [-0.14, 1.19] | 0.944 | 0.904 | 0.41 | 5643.7 | 1.000 |
| HF \| Age [+1 SD] | 0.08 | [-0.11, 0.25] | 0.801 | 0.390 | 0.04 | 5452.0 | 1.000 |
| HF \| Systolic BP [+1 SD] | 0.02 | [-0.16, 0.2] | 0.606 | 0.199 | 0.03 | 6035.2 | 1.000 |
| HF \| Diastolic BP [+1 SD] | 0.03 | [-0.21, 0.25] | 0.592 | 0.265 | 0.04 | 4869.7 | 1.000 |
| HF \| BMI [+1 SD] | 0.12 | [-0.25, 0.48] | 0.735 | 0.538 | 0.08 | 4323.4 | 1.000 |
| HF \| Total fat [+1 SD] | -0.26 | [-0.74, 0.2] | 0.863 | 0.750 | 0.14 | 4201.8 | 1.000 |
| HF \| Total muscle [+1 SD] | -0.09 | [-0.42, 0.27] | 0.693 | 0.480 | 0.07 | 4768.0 | 1.000 |
| HF \| (SFT) TMST [+1 SD] | -0.14 | [-0.36, 0.08] | 0.900 | 0.637 | 0.08 | 5852.0 | 1.000 |
| HF \| (SFT) TUG [+1 SD] | -0.14 | [-0.34, 0.07] | 0.908 | 0.652 | 0.08 | 5436.4 | 1.000 |
| HF \| (SFT) CSTS [+1 SD] | -0.03 | [-0.23, 0.17] | 0.625 | 0.245 | 0.04 | 6283.2 | 1.000 |
| LF \| Time-to-exercise [Peri] | -0.61 | [-0.89, -0.31] | 1.000 | 1.000 | 7889.24 | 9723.8 | 1.000 |
| LF \| Time-to-exercise [Post] | 0.32 | [0.02, 0.6] | 0.986 | 0.934 | 0.51 | 9124.7 | 1.000 |
| LF \| Sex [Female] | 0.59 | [-0.01, 1.14] | 0.978 | 0.954 | 0.73 | 7062.6 | 1.000 |
| LF \| Age [+1 SD] | 0.15 | [0.01, 0.31] | 0.981 | 0.760 | 0.21 | 8026.1 | 1.000 |
| LF \| Systolic BP [+1 SD] | -0.08 | [-0.23, 0.07] | 0.860 | 0.409 | 0.05 | 8105.3 | 1.000 |
| LF \| Diastolic BP [+1 SD] | 0.21 | [0.02, 0.4] | 0.986 | 0.877 | 0.38 | 4873.7 | 1.000 |
| LF \| BMI [+1 SD] | 0.20 | [-0.13, 0.49] | 0.897 | 0.735 | 0.12 | 4336.4 | 1.000 |
| LF \| Total fat [+1 SD] | -0.40 | [-0.79, 0] | 0.975 | 0.929 | 0.45 | 4025.2 | 1.000 |
| LF \| Total muscle [+1 SD] | -0.12 | [-0.41, 0.17] | 0.783 | 0.545 | 0.07 | 4343.4 | 1.000 |
| LF \| (SFT) TMST [+1 SD] | -0.11 | [-0.28, 0.08] | 0.874 | 0.528 | 0.06 | 6309.3 | 1.001 |
| LF \| (SFT) TUG [+1 SD] | -0.20 | [-0.36, -0.03] | 0.992 | 0.887 | 0.51 | 5564.9 | 1.000 |
| LF \| (SFT) CSTS [+1 SD] | -0.04 | [-0.21, 0.13] | 0.697 | 0.258 | 0.03 | 7957.7 | 1.001 |
| VLF \| Time-to-exercise [Peri] | -0.45 | [-0.75, -0.16] | 0.998 | 0.989 | 3.41 | 10687.2 | 1.000 |
| VLF \| Time-to-exercise [Post] | 0.17 | [-0.13, 0.47] | 0.873 | 0.686 | 0.10 | 10062.4 | 1.000 |
| VLF \| Sex [Female] | 0.57 | [0.02, 1.19] | 0.975 | 0.942 | 0.60 | 7769.8 | 1.000 |
| VLF \| Age [+1 SD] | 0.13 | [-0.02, 0.29] | 0.945 | 0.637 | 0.10 | 8508.3 | 1.000 |
| VLF \| Systolic BP [+1 SD] | 0.05 | [-0.11, 0.21] | 0.720 | 0.251 | 0.03 | 7826.3 | 1.000 |
| VLF \| Diastolic BP [+1 SD] | 0.18 | [-0.02, 0.38] | 0.960 | 0.773 | 0.17 | 5190.1 | 1.000 |
| VLF \| BMI [+1 SD] | 0.02 | [-0.31, 0.35] | 0.553 | 0.322 | 0.06 | 4320.5 | 1.000 |
| VLF \| Total fat [+1 SD] | -0.16 | [-0.58, 0.27] | 0.770 | 0.605 | 0.09 | 3985.6 | 1.000 |
| VLF \| Total muscle [+1 SD] | -0.01 | [-0.33, 0.29] | 0.517 | 0.286 | 0.05 | 4479.7 | 1.000 |
| VLF \| (SFT) TMST [+1 SD] | -0.19 | [-0.38, 0] | 0.976 | 0.833 | 0.23 | 6737.9 | 1.000 |
| VLF \| (SFT) TUG [+1 SD] | -0.14 | [-0.32, 0.03] | 0.944 | 0.683 | 0.10 | 6222.7 | 1.000 |
| VLF \| (SFT) CSTS [+1 SD] | 0.01 | [-0.16, 0.19] | 0.566 | 0.167 | 0.03 | 7835.1 | 1.000 |

**Table S4**. Model estimates and 95% credible interval based on highest density interval (HDI) from the posterior distribution on composite-domain parameters from the HRV components. These model effects are adjusted for confounders but not for baseline immune profile. pd, probability of direction; ps, probability of significance; BF, Bayes Factor in favor of the alternative hypothesis; ESS, effective sample size; R-hat, Potential reduction scale factor.

| Variable | Standardized Effect | 95% HDI | pd | ps | BF | ESS | R-hat |
| --- | --- | --- | --- | --- | --- | --- | --- |
| PNS index \| Intercept | -0.04 | [-0.52, 0.45] | 0.569 | 0.408 | 0.08 | 14156.4 | 1.000 |
| SNS index \| Intercept | -0.38 | [-0.71, -0.06] | 0.988 | 0.953 | 0.75 | 7722.3 | 1.001 |
| Stress index \| Intercept | -0.11 | [-0.48, 0.24] | 0.718 | 0.517 | 0.07 | 8801.8 | 1.001 |
| PNS index \| Time-to-exercise [Peri] | -0.08 | [-0.39, 0.25] | 0.688 | 0.455 | 0.06 | 19285.3 | 1.000 |
| PNS index \| Time-to-exercise [Post] | -0.07 | [-0.39, 0.25] | 0.664 | 0.421 | 0.06 | 18270.9 | 1.000 |
| PNS index \| Sex [Female] | 0.13 | [-0.52, 0.75] | 0.653 | 0.536 | 0.12 | 12565.3 | 1.000 |
| PNS index \| Age [+1 SD] | -0.11 | [-0.27, 0.06] | 0.907 | 0.560 | 0.07 | 15114.6 | 1.000 |
| PNS index \| Systolic BP [+1 SD] | -0.09 | [-0.25, 0.08] | 0.846 | 0.428 | 0.05 | 13717.2 | 1.000 |
| PNS index \| Diastolic BP [+1 SD] | -0.09 | [-0.3, 0.12] | 0.796 | 0.450 | 0.05 | 9862.9 | 1.000 |
| PNS index \| BMI [+1 SD] | 0.14 | [-0.2, 0.48] | 0.787 | 0.588 | 0.08 | 7875.7 | 1.000 |
| PNS index \| Total fat [+1 SD] | -0.16 | [-0.58, 0.28] | 0.762 | 0.606 | 0.10 | 7308.6 | 1.000 |
| PNS index \| Total muscle [+1 SD] | -0.10 | [-0.41, 0.22] | 0.726 | 0.504 | 0.07 | 9528.0 | 1.001 |
| PNS index \| (SFT) TMST [+1 SD] | -0.05 | [-0.25, 0.15] | 0.696 | 0.322 | 0.04 | 13866.4 | 1.000 |
| PNS index \| (SFT) TUG [+1 SD] | 0.01 | [-0.17, 0.19] | 0.517 | 0.154 | 0.03 | 11967.3 | 1.000 |
| PNS index \| (SFT) CSTS [+1 SD] | 0.17 | [-0.01, 0.36] | 0.969 | 0.783 | 0.17 | 14277.5 | 1.000 |
| SNS index \| Time-to-exercise [Peri] | 1.51 | [1.29, 1.72] | 1.000 | 1.000 | 5878.42 | 10270.6 | 1.000 |
| SNS index \| Time-to-exercise [Post] | 0.10 | [-0.11, 0.31] | 0.819 | 0.494 | 0.06 | 9636.6 | 1.000 |
| SNS index \| Sex [Female] | -0.24 | [-0.65, 0.2] | 0.861 | 0.729 | 0.12 | 7465.9 | 1.001 |
| SNS index \| Age [+1 SD] | -0.06 | [-0.17, 0.06] | 0.860 | 0.252 | 0.04 | 8072.3 | 1.000 |
| SNS index \| Systolic BP [+1 SD] | -0.03 | [-0.14, 0.08] | 0.699 | 0.105 | 0.02 | 8897.8 | 1.000 |
| SNS index \| Diastolic BP [+1 SD] | 0.06 | [-0.08, 0.2] | 0.818 | 0.309 | 0.04 | 5461.1 | 1.000 |
| SNS index \| BMI [+1 SD] | -0.12 | [-0.35, 0.11] | 0.846 | 0.560 | 0.07 | 4437.0 | 1.000 |
| SNS index \| Total fat [+1 SD] | 0.19 | [-0.12, 0.48] | 0.892 | 0.715 | 0.11 | 4343.6 | 1.001 |
| SNS index \| Total muscle [+1 SD] | 0.06 | [-0.15, 0.29] | 0.709 | 0.364 | 0.04 | 5382.1 | 1.000 |
| SNS index \| (SFT) TMST [+1 SD] | 0.09 | [-0.05, 0.23] | 0.895 | 0.419 | 0.05 | 8156.9 | 1.000 |
| SNS index \| (SFT) TUG [+1 SD] | 0.07 | [-0.05, 0.2] | 0.872 | 0.346 | 0.04 | 6418.4 | 1.000 |
| SNS index \| (SFT) CSTS [+1 SD] | 0.04 | [-0.09, 0.16] | 0.730 | 0.168 | 0.03 | 8056.9 | 1.000 |
| Stress index \| Time-to-exercise [Peri] | 1.23 | [0.98, 1.48] | 1.000 | 1.000 | 6638.90 | 9879.0 | 1.000 |
| Stress index \| Time-to-exercise [Post] | -0.08 | [-0.34, 0.16] | 0.746 | 0.446 | 0.05 | 9792.6 | 1.000 |
| Stress index \| Sex [Female] | -0.43 | [-0.9, 0.03] | 0.965 | 0.917 | 0.38 | 7753.7 | 1.001 |
| Stress index \| Age [+1 SD] | -0.11 | [-0.23, 0.02] | 0.958 | 0.560 | 0.09 | 8971.2 | 1.000 |
| Stress index \| Systolic BP [+1 SD] | -0.01 | [-0.13, 0.12] | 0.590 | 0.088 | 0.02 | 9783.6 | 1.000 |
| Stress index \| Diastolic BP [+1 SD] | -0.03 | [-0.18, 0.13] | 0.626 | 0.179 | 0.03 | 6038.6 | 1.000 |
| Stress index \| BMI [+1 SD] | -0.17 | [-0.42, 0.08] | 0.904 | 0.703 | 0.10 | 4706.6 | 1.001 |
| Stress index \| Total fat [+1 SD] | 0.29 | [-0.04, 0.61] | 0.961 | 0.875 | 0.26 | 4564.1 | 1.000 |
| Stress index \| Total muscle [+1 SD] | 0.07 | [-0.17, 0.32] | 0.728 | 0.418 | 0.05 | 5571.0 | 1.000 |
| Stress index \| (SFT) TMST [+1 SD] | 0.06 | [-0.09, 0.21] | 0.790 | 0.304 | 0.03 | 8888.2 | 1.000 |
| Stress index \| (SFT) TUG [+1 SD] | 0.14 | [0, 0.29] | 0.977 | 0.737 | 0.17 | 7180.0 | 1.000 |
| Stress index \| (SFT) CSTS [+1 SD] | 0.06 | [-0.09, 0.19] | 0.787 | 0.267 | 0.03 | 9217.7 | 1.000 |

## Considering immune cell influence

**Table S5**. Model estimates and 95% credible interval based on highest density interval (HDI) from the posterior distribution on time-domain parameters from the HRV components. These model effects are adjusted for confounders and for baseline immune profile. pd, probability of direction; ps, probability of significance; BF, Bayes Factor in favor of the alternative hypothesis; ESS, effective sample size; R-hat, Potential reduction scale factor.

| Variable | Standardized Effect | 95% HDI | pd | ps | BF | ESS | R-hat |
| --- | --- | --- | --- | --- | --- | --- | --- |
| RMSSD \| Intercept | -0.06 | [-0.5, 0.4] | 0.607 | 0.425 | 0.08 | 11746.5 | 1.000 |
| SDNN \| Intercept | -0.25 | [-0.68, 0.16] | 0.878 | 0.756 | 0.14 | 13190.1 | 1.000 |
| Mean R-R \| Intercept | 0.76 | [0.27, 1.24] | 0.999 | 0.995 | 6.17 | 8230.6 | 1.000 |
| RMSSD \| Time-to-exercise [Peri] | -1.09 | [-1.31, -0.86] | 1.000 | 1.000 | 5430.67 | 15910.9 | 1.000 |
| RMSSD \| Time-to-exercise [Post] | 0.00 | [-0.23, 0.23] | 0.481 | 0.179 | 0.04 | 16703.8 | 1.000 |
| RMSSD \| Total lymphocytes | -0.50 | [-1.98, 0.94] | 0.750 | 0.704 | 0.30 | 4947.1 | 1.001 |
| RMSSD \| B lymphocytes | 0.35 | [-0.11, 0.82] | 0.930 | 0.857 | 0.24 | 8062.3 | 1.000 |
| RMSSD \| T lymphocytes | 0.53 | [-0.85, 1.93] | 0.768 | 0.722 | 0.30 | 5161.5 | 1.001 |
| RMSSD \| T CD4+ lymphocytes | 0.19 | [-0.66, 1.07] | 0.663 | 0.580 | 0.16 | 6161.4 | 1.000 |
| RMSSD \| T CD8+ lymphocytes | -0.41 | [-1.02, 0.12] | 0.925 | 0.860 | 0.26 | 6654.7 | 1.000 |
| RMSSD \| NK lymphocytes | 0.08 | [-0.58, 0.76] | 0.592 | 0.476 | 0.12 | 4097.1 | 1.001 |
| RMSSD \| CD4+/CD8+ ratio | -0.58 | [-0.96, -0.2] | 0.998 | 0.994 | 4.89 | 7683.6 | 1.000 |
| RMSSD \| CD56^bright NK lymphocytes | -0.05 | [-0.53, 0.42] | 0.579 | 0.410 | 0.08 | 4933.9 | 1.001 |
| RMSSD \| CD56^dim NK lymphocytes | -0.10 | [-0.5, 0.32] | 0.688 | 0.503 | 0.08 | 4498.7 | 1.001 |
| RMSSD \| Sex [Female] | 0.66 | [0.03, 1.28] | 0.981 | 0.960 | 0.90 | 11470.1 | 1.000 |
| RMSSD \| Age [+1 SD] | 0.09 | [-0.09, 0.27] | 0.835 | 0.450 | 0.05 | 10883.0 | 1.001 |
| RMSSD \| Systolic BP [+1 SD] | -0.06 | [-0.24, 0.11] | 0.764 | 0.330 | 0.04 | 11194.3 | 1.000 |
| RMSSD \| Diastolic BP [+1 SD] | 0.05 | [-0.16, 0.26] | 0.679 | 0.309 | 0.04 | 8450.9 | 1.000 |
| RMSSD \| BMI [+1 SD] | 0.23 | [-0.12, 0.55] | 0.912 | 0.778 | 0.14 | 7525.8 | 1.000 |
| RMSSD \| Total fat [+1 SD] | -0.42 | [-0.84, 0.01] | 0.973 | 0.927 | 0.42 | 7179.8 | 1.001 |
| RMSSD \| Total muscle [+1 SD] | -0.09 | [-0.41, 0.23] | 0.701 | 0.472 | 0.06 | 8466.4 | 1.000 |
| RMSSD \| (SFT) TMST [+1 SD] | -0.10 | [-0.3, 0.1] | 0.831 | 0.502 | 0.06 | 10618.2 | 1.000 |
| RMSSD \| (SFT) TUG [+1 SD] | -0.05 | [-0.25, 0.14] | 0.718 | 0.319 | 0.04 | 10251.3 | 1.000 |
| RMSSD \| (SFT) CSTS [+1 SD] | 0.09 | [-0.1, 0.29] | 0.829 | 0.472 | 0.05 | 11449.3 | 1.000 |
| RMSSD \| Time-to-exercise [Peri]:Total lymphocytes | 0.21 | [-1.59, 1.86] | 0.596 | 0.550 | 0.30 | 5232.0 | 1.000 |
| RMSSD \| Time-to-exercise [Post]:Total lymphocytes | 0.22 | [-1.42, 1.99] | 0.598 | 0.553 | 0.30 | 5373.2 | 1.000 |
| RMSSD \| Time-to-exercise [Peri]:B lymphocytes | -0.39 | [-0.94, 0.15] | 0.925 | 0.859 | 0.26 | 8676.8 | 1.000 |
| RMSSD \| Time-to-exercise [Post]:B lymphocytes | -0.05 | [-0.6, 0.5] | 0.580 | 0.439 | 0.10 | 9019.3 | 1.000 |
| RMSSD \| Time-to-exercise [Peri]:T lymphocytes | -0.25 | [-1.93, 1.39] | 0.617 | 0.568 | 0.29 | 5542.9 | 1.000 |
| RMSSD \| Time-to-exercise [Post]:T lymphocytes | -0.04 | [-1.66, 1.64] | 0.518 | 0.465 | 0.28 | 5460.3 | 1.001 |
| RMSSD \| Time-to-exercise [Peri]:T CD4+ lymphocytes | -0.23 | [-1.32, 0.7] | 0.673 | 0.596 | 0.19 | 6317.5 | 1.000 |
| RMSSD \| Time-to-exercise [Post]:T CD4+ lymphocytes | -0.14 | [-1.12, 0.86] | 0.614 | 0.538 | 0.18 | 6572.0 | 1.000 |
| RMSSD \| Time-to-exercise [Peri]:T CD8+ lymphocytes | 0.53 | [-0.09, 1.25] | 0.943 | 0.900 | 0.39 | 6980.2 | 1.000 |
| RMSSD \| Time-to-exercise [Post]:T CD8+ lymphocytes | 0.20 | [-0.47, 0.86] | 0.728 | 0.620 | 0.14 | 7162.0 | 1.000 |
| RMSSD \| Time-to-exercise [Peri]:NK lymphocytes | 0.09 | [-0.73, 0.86] | 0.590 | 0.489 | 0.14 | 4312.8 | 1.000 |
| RMSSD \| Time-to-exercise [Post]:NK lymphocytes | -0.11 | [-0.93, 0.65] | 0.610 | 0.511 | 0.14 | 4414.9 | 1.001 |
| RMSSD \| Time-to-exercise [Peri]:CD4+/CD8+ ratio | 0.56 | [0.14, 1] | 0.995 | 0.982 | 1.69 | 8821.3 | 1.000 |
| RMSSD \| Time-to-exercise [Post]:CD4+/CD8+ ratio | 0.32 | [-0.1, 0.77] | 0.930 | 0.846 | 0.22 | 9093.2 | 1.000 |
| RMSSD \| Time-to-exercise [Peri]:CD56^bright NK lymphocytes | -0.06 | [-0.65, 0.48] | 0.582 | 0.449 | 0.10 | 5090.2 | 1.000 |
| RMSSD \| Time-to-exercise [Post]:CD56^bright NK lymphocytes | 0.06 | [-0.51, 0.63] | 0.587 | 0.447 | 0.10 | 5135.6 | 1.001 |
| RMSSD \| Time-to-exercise [Peri]:CD56^dim NK lymphocytes | 0.14 | [-0.36, 0.62] | 0.712 | 0.562 | 0.09 | 5096.5 | 1.000 |
| RMSSD \| Time-to-exercise [Post]:CD56^dim NK lymphocytes | -0.14 | [-0.62, 0.36] | 0.709 | 0.557 | 0.10 | 5147.4 | 1.001 |
| SDNN \| Time-to-exercise [Peri] | -1.00 | [-1.24, -0.77] | 1.000 | 1.000 | 6097.77 | 13914.3 | 1.000 |
| SDNN \| Time-to-exercise [Post] | 0.26 | [0.03, 0.5] | 0.986 | 0.918 | 0.43 | 14414.0 | 1.000 |
| SDNN \| Total lymphocytes | 0.47 | [-0.93, 1.94] | 0.738 | 0.693 | 0.30 | 4934.3 | 1.000 |
| SDNN \| B lymphocytes | -0.13 | [-0.58, 0.31] | 0.718 | 0.559 | 0.09 | 7514.8 | 1.000 |
| SDNN \| T lymphocytes | -0.50 | [-1.81, 0.86] | 0.763 | 0.715 | 0.29 | 5130.1 | 1.001 |
| SDNN \| T CD4+ lymphocytes | 0.30 | [-0.54, 1.12] | 0.759 | 0.682 | 0.19 | 5856.9 | 1.000 |
| SDNN \| T CD8+ lymphocytes | -0.14 | [-0.72, 0.39] | 0.695 | 0.561 | 0.11 | 6750.3 | 1.000 |
| SDNN \| NK lymphocytes | -0.08 | [-0.74, 0.59] | 0.594 | 0.477 | 0.12 | 4284.6 | 1.000 |
| SDNN \| CD4+/CD8+ ratio | -0.41 | [-0.78, -0.05] | 0.986 | 0.950 | 0.69 | 7989.6 | 1.000 |
| SDNN \| CD56^bright NK lymphocytes | -0.16 | [-0.62, 0.3] | 0.751 | 0.600 | 0.10 | 4965.5 | 1.000 |
| SDNN \| CD56^dim NK lymphocytes | -0.04 | [-0.45, 0.36] | 0.565 | 0.379 | 0.07 | 4719.1 | 1.000 |
| SDNN \| Sex [Female] | 0.76 | [0.19, 1.33] | 0.996 | 0.988 | 2.83 | 13025.3 | 1.000 |
| SDNN \| Age [+1 SD] | 0.17 | [0, 0.33] | 0.975 | 0.780 | 0.17 | 12834.7 | 1.000 |
| SDNN \| Systolic BP [+1 SD] | -0.06 | [-0.22, 0.1] | 0.784 | 0.325 | 0.04 | 13144.9 | 1.000 |
| SDNN \| Diastolic BP [+1 SD] | 0.18 | [-0.02, 0.36] | 0.966 | 0.794 | 0.17 | 10108.5 | 1.000 |
| SDNN \| BMI [+1 SD] | 0.25 | [-0.06, 0.56] | 0.942 | 0.825 | 0.17 | 8440.8 | 1.000 |
| SDNN \| Total fat [+1 SD] | -0.44 | [-0.84, -0.03] | 0.984 | 0.950 | 0.62 | 7962.8 | 1.000 |
| SDNN \| Total muscle [+1 SD] | -0.07 | [-0.39, 0.21] | 0.687 | 0.430 | 0.06 | 9102.3 | 1.000 |
| SDNN \| (SFT) TMST [+1 SD] | -0.11 | [-0.31, 0.07] | 0.879 | 0.540 | 0.07 | 11895.9 | 1.000 |
| SDNN \| (SFT) TUG [+1 SD] | -0.17 | [-0.35, -0.01] | 0.977 | 0.800 | 0.23 | 11015.4 | 1.000 |
| SDNN \| (SFT) CSTS [+1 SD] | 0.05 | [-0.12, 0.24] | 0.717 | 0.295 | 0.04 | 14393.2 | 1.000 |
| SDNN \| Time-to-exercise [Peri]:Total lymphocytes | -0.45 | [-2.18, 1.38] | 0.694 | 0.655 | 0.34 | 5472.2 | 1.000 |
| SDNN \| Time-to-exercise [Post]:Total lymphocytes | -1.18 | [-2.97, 0.55] | 0.906 | 0.886 | 0.71 | 5694.0 | 1.000 |
| SDNN \| Time-to-exercise [Peri]:B lymphocytes | 0.29 | [-0.27, 0.86] | 0.848 | 0.751 | 0.16 | 8415.9 | 1.000 |
| SDNN \| Time-to-exercise [Post]:B lymphocytes | 0.25 | [-0.3, 0.82] | 0.808 | 0.698 | 0.14 | 8365.5 | 1.000 |
| SDNN \| Time-to-exercise [Peri]:T lymphocytes | 0.74 | [-0.93, 2.48] | 0.799 | 0.764 | 0.41 | 5519.2 | 1.000 |
| SDNN \| Time-to-exercise [Post]:T lymphocytes | 0.56 | [-1.12, 2.28] | 0.745 | 0.707 | 0.36 | 5413.8 | 1.000 |
| SDNN \| Time-to-exercise [Peri]:T CD4+ lymphocytes | -0.58 | [-1.6, 0.45] | 0.868 | 0.822 | 0.33 | 6405.4 | 1.000 |
| SDNN \| Time-to-exercise [Post]:T CD4+ lymphocytes | 0.38 | [-0.63, 1.4] | 0.768 | 0.705 | 0.23 | 6405.9 | 1.000 |
| SDNN \| Time-to-exercise [Peri]:T CD8+ lymphocytes | 0.45 | [-0.26, 1.12] | 0.894 | 0.836 | 0.26 | 7335.1 | 1.000 |
| SDNN \| Time-to-exercise [Post]:T CD8+ lymphocytes | 0.30 | [-0.38, 0.98] | 0.806 | 0.721 | 0.17 | 7294.1 | 1.000 |
| SDNN \| Time-to-exercise [Peri]:NK lymphocytes | 0.04 | [-0.75, 0.91] | 0.540 | 0.443 | 0.14 | 4674.6 | 1.000 |
| SDNN \| Time-to-exercise [Post]:NK lymphocytes | 0.24 | [-0.59, 1.03] | 0.720 | 0.634 | 0.17 | 4594.3 | 1.001 |
| SDNN \| Time-to-exercise [Peri]:CD4+/CD8+ ratio | 0.64 | [0.2, 1.1] | 0.997 | 0.991 | 3.57 | 8965.8 | 1.000 |
| SDNN \| Time-to-exercise [Post]:CD4+/CD8+ ratio | 0.07 | [-0.37, 0.53] | 0.617 | 0.443 | 0.08 | 8838.5 | 1.000 |
| SDNN \| Time-to-exercise [Peri]:CD56^bright NK lymphocytes | 0.00 | [-0.59, 0.59] | 0.509 | 0.383 | 0.10 | 5262.0 | 1.000 |
| SDNN \| Time-to-exercise [Post]:CD56^bright NK lymphocytes | 0.08 | [-0.52, 0.67] | 0.608 | 0.472 | 0.10 | 5305.3 | 1.001 |
| SDNN \| Time-to-exercise [Peri]:CD56^dim NK lymphocytes | -0.12 | [-0.61, 0.4] | 0.671 | 0.521 | 0.09 | 5300.3 | 1.000 |
| SDNN \| Time-to-exercise [Post]:CD56^dim NK lymphocytes | -0.02 | [-0.52, 0.48] | 0.533 | 0.380 | 0.09 | 5101.2 | 1.001 |
| Mean R-R \| Time-to-exercise [Peri] | -1.62 | [-1.78, -1.47] | 1.000 | 1.000 | 3468.67 | 25550.2 | 1.000 |
| Mean R-R \| Time-to-exercise [Post] | -0.39 | [-0.55, -0.25] | 1.000 | 1.000 | 4229.17 | 25626.7 | 1.000 |
| Mean R-R \| Total lymphocytes | -1.20 | [-2.65, 0.25] | 0.951 | 0.933 | 0.91 | 5152.8 | 1.000 |
| Mean R-R \| B lymphocytes | -0.23 | [-0.66, 0.2] | 0.848 | 0.722 | 0.13 | 7741.2 | 1.000 |
| Mean R-R \| T lymphocytes | 1.16 | [-0.27, 2.51] | 0.946 | 0.930 | 0.88 | 6181.3 | 1.000 |
| Mean R-R \| T CD4+ lymphocytes | -0.17 | [-0.99, 0.7] | 0.661 | 0.570 | 0.15 | 6852.1 | 1.000 |
| Mean R-R \| T CD8+ lymphocytes | -0.12 | [-0.67, 0.41] | 0.673 | 0.536 | 0.10 | 8567.8 | 1.000 |
| Mean R-R \| NK lymphocytes | 0.37 | [-0.29, 1.03] | 0.873 | 0.799 | 0.22 | 5097.1 | 1.000 |
| Mean R-R \| CD4+/CD8+ ratio | -0.04 | [-0.4, 0.3] | 0.596 | 0.377 | 0.06 | 8627.3 | 1.000 |
| Mean R-R \| CD56^bright NK lymphocytes | -0.24 | [-0.67, 0.21] | 0.852 | 0.725 | 0.13 | 6352.8 | 1.000 |
| Mean R-R \| CD56^dim NK lymphocytes | 0.42 | [0.02, 0.81] | 0.981 | 0.945 | 0.56 | 5759.8 | 1.000 |
| Mean R-R \| Sex [Female] | -0.17 | [-0.86, 0.51] | 0.690 | 0.581 | 0.13 | 7823.3 | 1.000 |
| Mean R-R \| Age [+1 SD] | 0.04 | [-0.15, 0.23] | 0.661 | 0.271 | 0.04 | 7997.3 | 1.000 |
| Mean R-R \| Systolic BP [+1 SD] | 0.11 | [-0.08, 0.29] | 0.875 | 0.527 | 0.06 | 8293.5 | 1.000 |
| Mean R-R \| Diastolic BP [+1 SD] | -0.11 | [-0.34, 0.12] | 0.842 | 0.548 | 0.06 | 7559.7 | 1.000 |
| Mean R-R \| BMI [+1 SD] | -0.10 | [-0.46, 0.27] | 0.699 | 0.491 | 0.07 | 6230.9 | 1.000 |
| Mean R-R \| Total fat [+1 SD] | 0.12 | [-0.36, 0.59] | 0.702 | 0.543 | 0.09 | 5806.3 | 1.000 |
| Mean R-R \| Total muscle [+1 SD] | 0.06 | [-0.3, 0.4] | 0.622 | 0.401 | 0.06 | 7008.4 | 1.000 |
| Mean R-R \| (SFT) TMST [+1 SD] | -0.08 | [-0.3, 0.14] | 0.765 | 0.429 | 0.05 | 7830.6 | 1.000 |
| Mean R-R \| (SFT) TUG [+1 SD] | -0.01 | [-0.21, 0.2] | 0.537 | 0.190 | 0.03 | 8105.6 | 1.000 |
| Mean R-R \| (SFT) CSTS [+1 SD] | -0.03 | [-0.24, 0.18] | 0.618 | 0.258 | 0.04 | 8160.0 | 1.000 |
| Mean R-R \| Time-to-exercise [Peri]:Total lymphocytes | 1.00 | [-0.21, 2.32] | 0.944 | 0.923 | 0.76 | 8227.2 | 1.000 |
| Mean R-R \| Time-to-exercise [Post]:Total lymphocytes | 1.08 | [-0.17, 2.34] | 0.953 | 0.935 | 0.85 | 7640.0 | 1.000 |
| Mean R-R \| Time-to-exercise [Peri]:B lymphocytes | 0.36 | [-0.02, 0.74] | 0.967 | 0.909 | 0.33 | 14125.6 | 1.000 |
| Mean R-R \| Time-to-exercise [Post]:B lymphocytes | 0.17 | [-0.21, 0.55] | 0.803 | 0.633 | 0.09 | 13891.9 | 1.000 |
| Mean R-R \| Time-to-exercise [Peri]:T lymphocytes | -0.77 | [-2.09, 0.41] | 0.893 | 0.861 | 0.45 | 8491.3 | 1.000 |
| Mean R-R \| Time-to-exercise [Post]:T lymphocytes | -0.49 | [-1.77, 0.69] | 0.783 | 0.730 | 0.27 | 8049.6 | 1.000 |
| Mean R-R \| Time-to-exercise [Peri]:T CD4+ lymphocytes | -0.05 | [-0.78, 0.64] | 0.552 | 0.441 | 0.12 | 10094.2 | 1.000 |
| Mean R-R \| Time-to-exercise [Post]:T CD4+ lymphocytes | -0.25 | [-0.93, 0.46] | 0.754 | 0.662 | 0.15 | 10170.0 | 1.000 |
| Mean R-R \| Time-to-exercise [Peri]:T CD8+ lymphocytes | 0.03 | [-0.42, 0.51] | 0.557 | 0.393 | 0.08 | 11897.1 | 1.000 |
| Mean R-R \| Time-to-exercise [Post]:T CD8+ lymphocytes | -0.23 | [-0.73, 0.22] | 0.831 | 0.707 | 0.13 | 12355.9 | 1.000 |
| Mean R-R \| Time-to-exercise [Peri]:NK lymphocytes | -0.39 | [-0.97, 0.16] | 0.911 | 0.842 | 0.23 | 7043.3 | 1.000 |
| Mean R-R \| Time-to-exercise [Post]:NK lymphocytes | -0.24 | [-0.8, 0.34] | 0.797 | 0.686 | 0.13 | 6724.3 | 1.000 |
| Mean R-R \| Time-to-exercise [Peri]:CD4+/CD8+ ratio | 0.15 | [-0.15, 0.45] | 0.839 | 0.629 | 0.08 | 14998.3 | 1.000 |
| Mean R-R \| Time-to-exercise [Post]:CD4+/CD8+ ratio | 0.00 | [-0.29, 0.29] | 0.511 | 0.262 | 0.05 | 15032.8 | 1.000 |
| Mean R-R \| Time-to-exercise [Peri]:CD56^bright NK lymphocytes | 0.19 | [-0.2, 0.59] | 0.833 | 0.678 | 0.11 | 8916.9 | 1.000 |
| Mean R-R \| Time-to-exercise [Post]:CD56^bright NK lymphocytes | -0.07 | [-0.46, 0.33] | 0.630 | 0.429 | 0.07 | 8790.4 | 1.000 |
| Mean R-R \| Time-to-exercise [Peri]:CD56^dim NK lymphocytes | -0.56 | [-0.9, -0.21] | 0.999 | 0.995 | 6.43 | 7971.6 | 1.000 |
| Mean R-R \| Time-to-exercise [Post]:CD56^dim NK lymphocytes | -0.29 | [-0.64, 0.05] | 0.951 | 0.864 | 0.23 | 7595.2 | 1.000 |

**Table S6**. Model estimates and 95% credible interval based on highest density interval (HDI) from the posterior distribution on frequency-domain parameters from the HRV components. These model effects are adjusted for confounders and for baseline immune profile. pd, probability of direction; ps, probability of significance; BF, Bayes Factor in favor of the alternative hypothesis; ESS, effective sample size; R-hat, Potential reduction scale factor.

| Variable | Standardized Effect | 95% HDI | pd | ps | BF | ESS | R-hat |
| --- | --- | --- | --- | --- | --- | --- | --- |
| HF \| Intercept | -0.01 | [-0.5, 0.49] | 0.520 | 0.363 | 0.08 | 10389.1 | 1.000 |
| LF \| Intercept | -0.28 | [-0.72, 0.18] | 0.887 | 0.779 | 0.16 | 12888.4 | 1.000 |
| VLF \| Intercept | -0.36 | [-0.81, 0.1] | 0.938 | 0.867 | 0.25 | 13082.2 | 1.000 |
| HF \| Time-to-exercise [Peri] | -1.03 | [-1.26, -0.79] | 1.000 | 1.000 | 5703.11 | 16342.3 | 1.000 |
| HF \| Time-to-exercise [Post] | 0.05 | [-0.18, 0.3] | 0.660 | 0.342 | 0.04 | 16724.4 | 1.000 |
| HF \| Total lymphocytes | -1.20 | [-2.68, 0.38] | 0.936 | 0.917 | 0.81 | 6137.1 | 1.000 |
| HF \| B lymphocytes | 0.58 | [0.07, 1.07] | 0.989 | 0.970 | 1.08 | 7105.0 | 1.000 |
| HF \| T lymphocytes | 0.59 | [-0.92, 2.1] | 0.779 | 0.738 | 0.35 | 5778.4 | 1.000 |
| HF \| T CD4+ lymphocytes | 0.42 | [-0.5, 1.34] | 0.820 | 0.755 | 0.23 | 6104.2 | 1.000 |
| HF \| T CD8+ lymphocytes | 0.00 | [-0.61, 0.6] | 0.496 | 0.369 | 0.10 | 7219.2 | 1.000 |
| HF \| NK lymphocytes | 0.07 | [-0.61, 0.82] | 0.574 | 0.467 | 0.12 | 5258.3 | 1.001 |
| HF \| CD4+/CD8+ ratio | -0.47 | [-0.87, -0.07] | 0.989 | 0.965 | 0.85 | 7643.4 | 1.000 |
| HF \| CD56^bright NK lymphocytes | 0.01 | [-0.5, 0.53] | 0.523 | 0.378 | 0.09 | 5710.0 | 1.001 |
| HF \| CD56^dim NK lymphocytes | -0.09 | [-0.54, 0.36] | 0.649 | 0.473 | 0.08 | 5706.6 | 1.001 |
| HF \| Sex [Female] | 0.53 | [-0.16, 1.22] | 0.936 | 0.895 | 0.38 | 10151.9 | 1.000 |
| HF \| Age [+1 SD] | 0.16 | [-0.03, 0.36] | 0.947 | 0.738 | 0.13 | 10207.8 | 1.000 |
| HF \| Systolic BP [+1 SD] | -0.05 | [-0.22, 0.15] | 0.682 | 0.283 | 0.04 | 10511.5 | 1.000 |
| HF \| Diastolic BP [+1 SD] | 0.07 | [-0.15, 0.31] | 0.728 | 0.386 | 0.05 | 7425.5 | 1.000 |
| HF \| BMI [+1 SD] | -0.02 | [-0.39, 0.34] | 0.544 | 0.333 | 0.06 | 6938.7 | 1.000 |
| HF \| Total fat [+1 SD] | -0.11 | [-0.58, 0.36] | 0.680 | 0.521 | 0.09 | 6575.9 | 1.000 |
| HF \| Total muscle [+1 SD] | 0.07 | [-0.26, 0.44] | 0.660 | 0.443 | 0.06 | 8244.0 | 1.000 |
| HF \| (SFT) TMST [+1 SD] | -0.08 | [-0.3, 0.14] | 0.773 | 0.436 | 0.05 | 9515.1 | 1.000 |
| HF \| (SFT) TUG [+1 SD] | -0.06 | [-0.27, 0.14] | 0.735 | 0.364 | 0.04 | 9537.5 | 1.000 |
| HF \| (SFT) CSTS [+1 SD] | 0.00 | [-0.2, 0.22] | 0.515 | 0.189 | 0.04 | 11410.0 | 1.000 |
| HF \| Time-to-exercise [Peri]:Total lymphocytes | 1.16 | [-0.59, 2.91] | 0.898 | 0.879 | 0.68 | 6861.5 | 1.000 |
| HF \| Time-to-exercise [Post]:Total lymphocytes | 0.75 | [-1, 2.56] | 0.800 | 0.768 | 0.44 | 6502.0 | 1.000 |
| HF \| Time-to-exercise [Peri]:B lymphocytes | -0.64 | [-1.21, -0.09] | 0.986 | 0.970 | 1.16 | 8057.7 | 1.000 |
| HF \| Time-to-exercise [Post]:B lymphocytes | -0.25 | [-0.81, 0.32] | 0.810 | 0.703 | 0.14 | 8610.1 | 1.000 |
| HF \| Time-to-exercise [Peri]:T lymphocytes | -0.54 | [-2.27, 1.22] | 0.730 | 0.690 | 0.35 | 6427.4 | 1.000 |
| HF \| Time-to-exercise [Post]:T lymphocytes | -0.67 | [-2.4, 1.1] | 0.771 | 0.739 | 0.41 | 5902.7 | 1.000 |
| HF \| Time-to-exercise [Peri]:T CD4+ lymphocytes | -0.38 | [-1.46, 0.63] | 0.766 | 0.704 | 0.23 | 7060.2 | 1.000 |
| HF \| Time-to-exercise [Post]:T CD4+ lymphocytes | 0.16 | [-0.88, 1.24] | 0.620 | 0.548 | 0.19 | 6515.6 | 1.001 |
| HF \| Time-to-exercise [Peri]:T CD8+ lymphocytes | 0.11 | [-0.58, 0.82] | 0.627 | 0.516 | 0.12 | 8094.5 | 1.000 |
| HF \| Time-to-exercise [Post]:T CD8+ lymphocytes | 0.28 | [-0.41, 1.01] | 0.788 | 0.696 | 0.16 | 7744.2 | 1.000 |
| HF \| Time-to-exercise [Peri]:NK lymphocytes | -0.02 | [-0.82, 0.81] | 0.516 | 0.419 | 0.14 | 5823.7 | 1.001 |
| HF \| Time-to-exercise [Post]:NK lymphocytes | -0.31 | [-1.14, 0.52] | 0.770 | 0.692 | 0.18 | 5274.4 | 1.000 |
| HF \| Time-to-exercise [Peri]:CD4+/CD8+ ratio | 0.53 | [0.09, 0.98] | 0.990 | 0.972 | 1.18 | 9830.6 | 1.000 |
| HF \| Time-to-exercise [Post]:CD4+/CD8+ ratio | 0.16 | [-0.31, 0.59] | 0.756 | 0.597 | 0.10 | 10053.8 | 1.000 |
| HF \| Time-to-exercise [Peri]:CD56^bright NK lymphocytes | -0.12 | [-0.74, 0.45] | 0.663 | 0.537 | 0.11 | 6483.9 | 1.000 |
| HF \| Time-to-exercise [Post]:CD56^bright NK lymphocytes | 0.22 | [-0.38, 0.82] | 0.765 | 0.652 | 0.13 | 5933.4 | 1.000 |
| HF \| Time-to-exercise [Peri]:CD56^dim NK lymphocytes | 0.08 | [-0.43, 0.59] | 0.621 | 0.469 | 0.09 | 5949.3 | 1.001 |
| HF \| Time-to-exercise [Post]:CD56^dim NK lymphocytes | -0.20 | [-0.72, 0.31] | 0.770 | 0.646 | 0.12 | 5828.7 | 1.000 |
| LF \| Time-to-exercise [Peri] | -0.66 | [-0.95, -0.38] | 1.000 | 1.000 | 105.99 | 13461.7 | 1.000 |
| LF \| Time-to-exercise [Post] | 0.29 | [0, 0.58] | 0.973 | 0.895 | 0.30 | 13873.6 | 1.000 |
| LF \| Total lymphocytes | 0.51 | [-1.03, 1.96] | 0.749 | 0.705 | 0.32 | 4789.9 | 1.000 |
| LF \| B lymphocytes | -0.43 | [-0.94, 0.07] | 0.953 | 0.902 | 0.37 | 6409.2 | 1.000 |
| LF \| T lymphocytes | -0.71 | [-2.15, 0.77] | 0.830 | 0.791 | 0.39 | 4626.7 | 1.000 |
| LF \| T CD4+ lymphocytes | 0.27 | [-0.67, 1.19] | 0.721 | 0.646 | 0.19 | 4740.2 | 1.000 |
| LF \| T CD8+ lymphocytes | 0.00 | [-0.6, 0.62] | 0.495 | 0.369 | 0.10 | 5471.5 | 1.000 |
| LF \| NK lymphocytes | 0.21 | [-0.5, 0.93] | 0.710 | 0.608 | 0.14 | 3864.5 | 1.000 |
| LF \| CD4+/CD8+ ratio | -0.27 | [-0.65, 0.17] | 0.897 | 0.783 | 0.16 | 6563.8 | 1.000 |
| LF \| CD56^bright NK lymphocytes | -0.29 | [-0.8, 0.25] | 0.857 | 0.753 | 0.16 | 4293.8 | 1.000 |
| LF \| CD56^dim NK lymphocytes | 0.16 | [-0.29, 0.61] | 0.762 | 0.602 | 0.10 | 4034.0 | 1.000 |
| LF \| Sex [Female] | 0.61 | [0.02, 1.2] | 0.979 | 0.956 | 0.79 | 12116.8 | 1.000 |
| LF \| Age [+1 SD] | 0.17 | [-0.01, 0.33] | 0.972 | 0.780 | 0.18 | 11854.4 | 1.000 |
| LF \| Systolic BP [+1 SD] | -0.09 | [-0.26, 0.07] | 0.872 | 0.472 | 0.06 | 12450.7 | 1.000 |
| LF \| Diastolic BP [+1 SD] | 0.21 | [0.02, 0.42] | 0.982 | 0.868 | 0.28 | 8842.9 | 1.000 |
| LF \| BMI [+1 SD] | 0.20 | [-0.12, 0.52] | 0.894 | 0.736 | 0.12 | 6972.0 | 1.000 |
| LF \| Total fat [+1 SD] | -0.36 | [-0.76, 0.05] | 0.959 | 0.896 | 0.31 | 6676.7 | 1.000 |
| LF \| Total muscle [+1 SD] | -0.07 | [-0.39, 0.24] | 0.680 | 0.426 | 0.06 | 7951.5 | 1.000 |
| LF \| (SFT) TMST [+1 SD] | -0.10 | [-0.29, 0.09] | 0.837 | 0.494 | 0.06 | 12364.6 | 1.000 |
| LF \| (SFT) TUG [+1 SD] | -0.19 | [-0.37, -0.02] | 0.984 | 0.849 | 0.34 | 10049.5 | 1.000 |
| LF \| (SFT) CSTS [+1 SD] | -0.01 | [-0.19, 0.18] | 0.535 | 0.164 | 0.03 | 12818.5 | 1.000 |
| LF \| Time-to-exercise [Peri]:Total lymphocytes | 0.05 | [-2.05, 2.04] | 0.516 | 0.476 | 0.34 | 5357.0 | 1.000 |
| LF \| Time-to-exercise [Post]:Total lymphocytes | -1.58 | [-3.65, 0.41] | 0.936 | 0.922 | 1.08 | 5721.8 | 1.000 |
| LF \| Time-to-exercise [Peri]:B lymphocytes | 0.44 | [-0.25, 1.11] | 0.904 | 0.841 | 0.27 | 6224.0 | 1.000 |
| LF \| Time-to-exercise [Post]:B lymphocytes | 0.50 | [-0.15, 1.21] | 0.926 | 0.877 | 0.33 | 7113.3 | 1.000 |
| LF \| Time-to-exercise [Peri]:T lymphocytes | 0.73 | [-1.4, 2.56] | 0.767 | 0.736 | 0.45 | 5192.4 | 1.000 |
| LF \| Time-to-exercise [Post]:T lymphocytes | 0.69 | [-1.35, 2.71] | 0.753 | 0.722 | 0.44 | 5172.2 | 1.000 |
| LF \| Time-to-exercise [Peri]:T CD4+ lymphocytes | -0.90 | [-2.13, 0.32] | 0.923 | 0.898 | 0.57 | 5110.7 | 1.000 |
| LF \| Time-to-exercise [Post]:T CD4+ lymphocytes | 0.53 | [-0.67, 1.75] | 0.799 | 0.754 | 0.30 | 5364.7 | 1.000 |
| LF \| Time-to-exercise [Peri]:T CD8+ lymphocytes | 0.49 | [-0.33, 1.32] | 0.881 | 0.824 | 0.27 | 5958.8 | 1.000 |
| LF \| Time-to-exercise [Post]:T CD8+ lymphocytes | 0.32 | [-0.51, 1.16] | 0.780 | 0.704 | 0.19 | 6284.9 | 1.000 |
| LF \| Time-to-exercise [Peri]:NK lymphocytes | -0.22 | [-1.2, 0.74] | 0.668 | 0.593 | 0.18 | 4432.4 | 1.000 |
| LF \| Time-to-exercise [Post]:NK lymphocytes | 0.02 | [-0.93, 1] | 0.514 | 0.433 | 0.16 | 4290.9 | 1.000 |
| LF \| Time-to-exercise [Peri]:CD4+/CD8+ ratio | 0.76 | [0.24, 1.33] | 0.997 | 0.991 | 3.39 | 7461.8 | 1.000 |
| LF \| Time-to-exercise [Post]:CD4+/CD8+ ratio | -0.05 | [-0.61, 0.49] | 0.576 | 0.429 | 0.09 | 7541.9 | 1.000 |
| LF \| Time-to-exercise [Peri]:CD56^bright NK lymphocytes | -0.04 | [-0.78, 0.66] | 0.545 | 0.439 | 0.13 | 4874.7 | 1.000 |
| LF \| Time-to-exercise [Post]:CD56^bright NK lymphocytes | 0.32 | [-0.4, 1.02] | 0.812 | 0.729 | 0.18 | 4629.8 | 1.000 |
| LF \| Time-to-exercise [Peri]:CD56^dim NK lymphocytes | -0.38 | [-0.99, 0.23] | 0.891 | 0.820 | 0.22 | 4364.1 | 1.000 |
| LF \| Time-to-exercise [Post]:CD56^dim NK lymphocytes | 0.05 | [-0.54, 0.66] | 0.563 | 0.429 | 0.10 | 4459.8 | 1.000 |
| VLF \| Time-to-exercise [Peri] | -0.47 | [-0.77, -0.17] | 0.999 | 0.993 | 9.02 | 14597.6 | 1.000 |
| VLF \| Time-to-exercise [Post] | 0.19 | [-0.11, 0.49] | 0.894 | 0.727 | 0.11 | 15238.9 | 1.000 |
| VLF \| Total lymphocytes | 2.15 | [0.61, 3.71] | 0.996 | 0.994 | 8.14 | 5523.0 | 1.000 |
| VLF \| B lymphocytes | -0.70 | [-1.22, -0.17] | 0.994 | 0.987 | 2.26 | 6860.2 | 1.000 |
| VLF \| T lymphocytes | -1.82 | [-3.3, -0.26] | 0.991 | 0.987 | 3.73 | 4569.3 | 1.000 |
| VLF \| T CD4+ lymphocytes | 0.13 | [-0.83, 1.1] | 0.607 | 0.526 | 0.17 | 4731.5 | 1.000 |
| VLF \| T CD8+ lymphocytes | -0.04 | [-0.67, 0.6] | 0.550 | 0.428 | 0.11 | 5582.0 | 1.000 |
| VLF \| NK lymphocytes | -0.69 | [-1.41, 0.07] | 0.965 | 0.941 | 0.66 | 4073.6 | 1.000 |
| VLF \| CD4+/CD8+ ratio | -0.13 | [-0.57, 0.28] | 0.728 | 0.557 | 0.09 | 7324.4 | 1.000 |
| VLF \| CD56^bright NK lymphocytes | 0.01 | [-0.53, 0.57] | 0.517 | 0.371 | 0.09 | 4372.1 | 1.000 |
| VLF \| CD56^dim NK lymphocytes | 0.07 | [-0.38, 0.56] | 0.610 | 0.451 | 0.09 | 4279.0 | 1.000 |
| VLF \| Sex [Female] | 0.65 | [0.05, 1.27] | 0.981 | 0.961 | 0.95 | 12462.4 | 1.000 |
| VLF \| Age [+1 SD] | 0.13 | [-0.05, 0.3] | 0.928 | 0.636 | 0.09 | 11682.3 | 1.000 |
| VLF \| Systolic BP [+1 SD] | 0.03 | [-0.13, 0.2] | 0.651 | 0.220 | 0.03 | 13458.1 | 1.000 |
| VLF \| Diastolic BP [+1 SD] | 0.18 | [-0.02, 0.38] | 0.960 | 0.784 | 0.16 | 8785.0 | 1.000 |
| VLF \| BMI [+1 SD] | 0.06 | [-0.26, 0.4] | 0.640 | 0.410 | 0.06 | 7517.9 | 1.000 |
| VLF \| Total fat [+1 SD] | -0.20 | [-0.63, 0.23] | 0.824 | 0.679 | 0.11 | 7117.6 | 1.000 |
| VLF \| Total muscle [+1 SD] | 0.00 | [-0.32, 0.31] | 0.497 | 0.268 | 0.05 | 8440.7 | 1.000 |
| VLF \| (SFT) TMST [+1 SD] | -0.17 | [-0.37, 0.03] | 0.944 | 0.736 | 0.12 | 12072.5 | 1.000 |
| VLF \| (SFT) TUG [+1 SD] | -0.07 | [-0.26, 0.1] | 0.777 | 0.390 | 0.04 | 10657.9 | 1.000 |
| VLF \| (SFT) CSTS [+1 SD] | 0.05 | [-0.15, 0.25] | 0.678 | 0.289 | 0.04 | 12021.7 | 1.000 |
| VLF \| Time-to-exercise [Peri]:Total lymphocytes | -0.93 | [-3.03, 1.13] | 0.813 | 0.786 | 0.52 | 6297.2 | 1.000 |
| VLF \| Time-to-exercise [Post]:Total lymphocytes | -1.10 | [-3.15, 0.97] | 0.849 | 0.828 | 0.62 | 6111.7 | 1.000 |
| VLF \| Time-to-exercise [Peri]:B lymphocytes | 0.88 | [0.18, 1.57] | 0.993 | 0.987 | 2.70 | 7814.4 | 1.000 |
| VLF \| Time-to-exercise [Post]:B lymphocytes | 0.25 | [-0.49, 0.91] | 0.766 | 0.672 | 0.16 | 7604.7 | 1.000 |
| VLF \| Time-to-exercise [Peri]:T lymphocytes | 1.11 | [-0.95, 3.15] | 0.858 | 0.833 | 0.61 | 5964.7 | 1.000 |
| VLF \| Time-to-exercise [Post]:T lymphocytes | 0.52 | [-1.53, 2.56] | 0.696 | 0.660 | 0.40 | 5840.7 | 1.000 |
| VLF \| Time-to-exercise [Peri]:T CD4+ lymphocytes | -0.42 | [-1.68, 0.85] | 0.742 | 0.691 | 0.27 | 5544.4 | 1.000 |
| VLF \| Time-to-exercise [Post]:T CD4+ lymphocytes | 0.48 | [-0.74, 1.74] | 0.775 | 0.727 | 0.28 | 5824.1 | 1.000 |
| VLF \| Time-to-exercise [Peri]:T CD8+ lymphocytes | 0.44 | [-0.41, 1.3] | 0.845 | 0.781 | 0.25 | 6395.2 | 1.000 |
| VLF \| Time-to-exercise [Post]:T CD8+ lymphocytes | 0.13 | [-0.75, 0.94] | 0.620 | 0.528 | 0.15 | 6567.2 | 1.000 |
| VLF \| Time-to-exercise [Peri]:NK lymphocytes | 0.15 | [-0.86, 1.15] | 0.615 | 0.533 | 0.18 | 4442.1 | 1.000 |
| VLF \| Time-to-exercise [Post]:NK lymphocytes | 0.59 | [-0.37, 1.6] | 0.880 | 0.837 | 0.34 | 4497.6 | 1.000 |
| VLF \| Time-to-exercise [Peri]:CD4+/CD8+ ratio | 0.46 | [-0.12, 1.02] | 0.941 | 0.892 | 0.34 | 8302.7 | 1.000 |
| VLF \| Time-to-exercise [Post]:CD4+/CD8+ ratio | -0.21 | [-0.77, 0.35] | 0.775 | 0.655 | 0.13 | 8473.4 | 1.000 |
| VLF \| Time-to-exercise [Peri]:CD56^bright NK lymphocytes | 0.02 | [-0.71, 0.77] | 0.518 | 0.416 | 0.12 | 4749.0 | 1.000 |
| VLF \| Time-to-exercise [Post]:CD56^bright NK lymphocytes | -0.10 | [-0.85, 0.61] | 0.607 | 0.502 | 0.13 | 4977.1 | 1.000 |
| VLF \| Time-to-exercise [Peri]:CD56^dim NK lymphocytes | -0.55 | [-1.17, 0.1] | 0.956 | 0.920 | 0.49 | 4649.6 | 1.000 |
| VLF \| Time-to-exercise [Post]:CD56^dim NK lymphocytes | -0.01 | [-0.61, 0.63] | 0.515 | 0.390 | 0.11 | 4756.2 | 1.000 |

**Table S7**. Model estimates and 95% credible interval based on highest density interval (HDI) from the posterior distribution on composite-domain parameters from the HRV components. These model effects are adjusted for confounders and for baseline immune profile. pd, probability of direction; ps, probability of significance; BF, Bayes Factor in favor of the alternative hypothesis; ESS, effective sample size; R-hat, Potential reduction scale factor.

| Variable | Standardized Effect | 95% HDI | pd | ps | BF | ESS | R-hat |
| --- | --- | --- | --- | --- | --- | --- | --- |
| PNS index \| Intercept | -0.05 | [-0.61, 0.46] | 0.581 | 0.434 | 0.09 | 13812.0 | 1.000 |
| SNS index \| Intercept | -0.36 | [-0.7, -0.02] | 0.981 | 0.935 | 0.51 | 7396.3 | 1.001 |
| Stress index \| Intercept | -0.10 | [-0.46, 0.3] | 0.692 | 0.498 | 0.08 | 8611.8 | 1.000 |
| PNS index \| Time-to-exercise [Peri] | -0.06 | [-0.41, 0.29] | 0.633 | 0.418 | 0.07 | 16628.0 | 1.000 |
| PNS index \| Time-to-exercise [Post] | -0.07 | [-0.41, 0.29] | 0.647 | 0.427 | 0.06 | 15780.3 | 1.000 |
| PNS index \| Total lymphocytes | 0.26 | [-1.52, 2.07] | 0.612 | 0.569 | 0.31 | 4710.8 | 1.001 |
| PNS index \| B lymphocytes | -0.08 | [-0.68, 0.52] | 0.605 | 0.478 | 0.10 | 6525.9 | 1.001 |
| PNS index \| T lymphocytes | 0.18 | [-1.57, 1.9] | 0.583 | 0.536 | 0.30 | 4399.8 | 1.001 |
| PNS index \| T CD4+ lymphocytes | -0.26 | [-1.36, 0.86] | 0.674 | 0.610 | 0.21 | 4516.1 | 1.000 |
| PNS index \| T CD8+ lymphocytes | -0.21 | [-0.94, 0.54] | 0.712 | 0.613 | 0.15 | 5688.2 | 1.000 |
| PNS index \| NK lymphocytes | -0.03 | [-0.84, 0.82] | 0.528 | 0.431 | 0.14 | 3530.0 | 1.002 |
| PNS index \| CD4+/CD8+ ratio | -0.03 | [-0.52, 0.47] | 0.547 | 0.389 | 0.09 | 6485.4 | 1.000 |
| PNS index \| CD56^bright NK lymphocytes | -0.02 | [-0.64, 0.6] | 0.525 | 0.397 | 0.11 | 3993.0 | 1.002 |
| PNS index \| CD56^dim NK lymphocytes | -0.02 | [-0.56, 0.5] | 0.533 | 0.390 | 0.09 | 3827.6 | 1.003 |
| PNS index \| Sex [Female] | 0.15 | [-0.56, 0.84] | 0.665 | 0.558 | 0.13 | 13160.9 | 1.001 |
| PNS index \| Age [+1 SD] | -0.12 | [-0.32, 0.08] | 0.874 | 0.570 | 0.06 | 13881.4 | 1.000 |
| PNS index \| Systolic BP [+1 SD] | -0.13 | [-0.32, 0.07] | 0.902 | 0.599 | 0.07 | 14470.2 | 1.000 |
| PNS index \| Diastolic BP [+1 SD] | -0.09 | [-0.33, 0.14] | 0.769 | 0.456 | 0.05 | 10467.4 | 1.000 |
| PNS index \| BMI [+1 SD] | 0.18 | [-0.18, 0.56] | 0.835 | 0.677 | 0.10 | 8178.6 | 1.001 |
| PNS index \| Total fat [+1 SD] | -0.20 | [-0.68, 0.28] | 0.797 | 0.658 | 0.11 | 7733.6 | 1.001 |
| PNS index \| Total muscle [+1 SD] | -0.13 | [-0.51, 0.22] | 0.766 | 0.578 | 0.08 | 9098.9 | 1.000 |
| PNS index \| (SFT) TMST [+1 SD] | -0.03 | [-0.26, 0.19] | 0.594 | 0.274 | 0.04 | 13142.4 | 1.000 |
| PNS index \| (SFT) TUG [+1 SD] | 0.01 | [-0.2, 0.22] | 0.532 | 0.194 | 0.04 | 12378.6 | 1.001 |
| PNS index \| (SFT) CSTS [+1 SD] | 0.16 | [-0.06, 0.37] | 0.928 | 0.706 | 0.10 | 14351.2 | 1.000 |
| PNS index \| Time-to-exercise [Peri]:Total lymphocytes | -0.42 | [-2.84, 1.97] | 0.636 | 0.604 | 0.43 | 5425.9 | 1.001 |
| PNS index \| Time-to-exercise [Post]:Total lymphocytes | 0.10 | [-2.28, 2.56] | 0.529 | 0.496 | 0.42 | 5746.1 | 1.000 |
| PNS index \| Time-to-exercise [Peri]:B lymphocytes | -0.33 | [-1.16, 0.48] | 0.784 | 0.704 | 0.18 | 7597.1 | 1.000 |
| PNS index \| Time-to-exercise [Post]:B lymphocytes | 0.00 | [-0.81, 0.8] | 0.497 | 0.402 | 0.14 | 7835.8 | 1.000 |
| PNS index \| Time-to-exercise [Peri]:T lymphocytes | -0.25 | [-2.56, 2.09] | 0.586 | 0.550 | 0.40 | 5295.8 | 1.001 |
| PNS index \| Time-to-exercise [Post]:T lymphocytes | 0.04 | [-2.35, 2.37] | 0.513 | 0.478 | 0.41 | 5774.7 | 1.001 |
| PNS index \| Time-to-exercise [Peri]:T CD4+ lymphocytes | 0.45 | [-1.03, 1.89] | 0.730 | 0.682 | 0.30 | 5641.1 | 1.000 |
| PNS index \| Time-to-exercise [Post]:T CD4+ lymphocytes | -0.08 | [-1.56, 1.37] | 0.543 | 0.491 | 0.25 | 5514.2 | 1.000 |
| PNS index \| Time-to-exercise [Peri]:T CD8+ lymphocytes | 0.41 | [-0.57, 1.42] | 0.792 | 0.734 | 0.24 | 7162.7 | 1.000 |
| PNS index \| Time-to-exercise [Post]:T CD8+ lymphocytes | -0.04 | [-1.05, 0.96] | 0.528 | 0.448 | 0.17 | 6836.5 | 1.000 |
| PNS index \| Time-to-exercise [Peri]:NK lymphocytes | 0.65 | [-0.56, 1.77] | 0.867 | 0.829 | 0.37 | 4146.7 | 1.002 |
| PNS index \| Time-to-exercise [Post]:NK lymphocytes | -0.04 | [-1.16, 1.13] | 0.528 | 0.461 | 0.20 | 4087.1 | 1.001 |
| PNS index \| Time-to-exercise [Peri]:CD4+/CD8+ ratio | -0.11 | [-0.76, 0.55] | 0.636 | 0.518 | 0.12 | 9541.3 | 1.000 |
| PNS index \| Time-to-exercise [Post]:CD4+/CD8+ ratio | 0.03 | [-0.61, 0.7] | 0.536 | 0.418 | 0.11 | 9192.6 | 1.000 |
| PNS index \| Time-to-exercise [Peri]:CD56^bright NK lymphocytes | -0.55 | [-1.41, 0.3] | 0.899 | 0.850 | 0.32 | 4523.8 | 1.002 |
| PNS index \| Time-to-exercise [Post]:CD56^bright NK lymphocytes | 0.01 | [-0.83, 0.86] | 0.508 | 0.414 | 0.14 | 4602.7 | 1.001 |
| PNS index \| Time-to-exercise [Peri]:CD56^dim NK lymphocytes | 0.64 | [-0.11, 1.35] | 0.955 | 0.926 | 0.55 | 4582.5 | 1.002 |
| PNS index \| Time-to-exercise [Post]:CD56^dim NK lymphocytes | -0.03 | [-0.75, 0.69] | 0.532 | 0.428 | 0.13 | 4393.1 | 1.001 |
| SNS index \| Time-to-exercise [Peri] | 1.53 | [1.32, 1.75] | 1.000 | 1.000 | 5923.81 | 10178.3 | 1.000 |
| SNS index \| Time-to-exercise [Post] | 0.10 | [-0.12, 0.32] | 0.819 | 0.509 | 0.06 | 10491.4 | 1.000 |
| SNS index \| Total lymphocytes | -0.06 | [-1.2, 1.11] | 0.544 | 0.476 | 0.20 | 2376.5 | 1.000 |
| SNS index \| B lymphocytes | -0.05 | [-0.46, 0.32] | 0.608 | 0.409 | 0.07 | 3776.4 | 1.000 |
| SNS index \| T lymphocytes | -0.31 | [-1.46, 0.76] | 0.708 | 0.644 | 0.22 | 2502.6 | 1.000 |
| SNS index \| T CD4+ lymphocytes | 0.20 | [-0.5, 0.89] | 0.713 | 0.606 | 0.14 | 2854.0 | 1.000 |
| SNS index \| T CD8+ lymphocytes | 0.32 | [-0.15, 0.78] | 0.911 | 0.823 | 0.20 | 3336.7 | 1.000 |
| SNS index \| NK lymphocytes | 0.10 | [-0.46, 0.63] | 0.635 | 0.498 | 0.10 | 2032.5 | 1.000 |
| SNS index \| CD4+/CD8+ ratio | 0.16 | [-0.15, 0.48] | 0.846 | 0.652 | 0.09 | 4447.2 | 1.001 |
| SNS index \| CD56^bright NK lymphocytes | 0.02 | [-0.37, 0.42] | 0.539 | 0.345 | 0.07 | 2646.3 | 1.000 |
| SNS index \| CD56^dim NK lymphocytes | 0.09 | [-0.23, 0.45] | 0.711 | 0.488 | 0.07 | 2428.3 | 1.000 |
| SNS index \| Sex [Female] | -0.25 | [-0.71, 0.19] | 0.861 | 0.742 | 0.14 | 6548.9 | 1.002 |
| SNS index \| Age [+1 SD] | -0.04 | [-0.17, 0.09] | 0.725 | 0.186 | 0.03 | 8358.7 | 1.000 |
| SNS index \| Systolic BP [+1 SD] | 0.00 | [-0.13, 0.12] | 0.488 | 0.055 | 0.02 | 8598.1 | 1.000 |
| SNS index \| Diastolic BP [+1 SD] | 0.01 | [-0.14, 0.16] | 0.573 | 0.131 | 0.03 | 6485.5 | 1.000 |
| SNS index \| BMI [+1 SD] | -0.02 | [-0.27, 0.22] | 0.575 | 0.274 | 0.04 | 4405.9 | 1.000 |
| SNS index \| Total fat [+1 SD] | 0.08 | [-0.23, 0.4] | 0.689 | 0.462 | 0.06 | 3877.2 | 1.001 |
| SNS index \| Total muscle [+1 SD] | -0.04 | [-0.29, 0.19] | 0.642 | 0.315 | 0.04 | 5171.8 | 1.000 |
| SNS index \| (SFT) TMST [+1 SD] | 0.10 | [-0.05, 0.25] | 0.915 | 0.523 | 0.07 | 7466.1 | 1.000 |
| SNS index \| (SFT) TUG [+1 SD] | 0.03 | [-0.11, 0.17] | 0.653 | 0.157 | 0.03 | 7392.9 | 1.001 |
| SNS index \| (SFT) CSTS [+1 SD] | -0.07 | [-0.22, 0.07] | 0.841 | 0.355 | 0.04 | 7632.4 | 1.001 |
| SNS index \| Time-to-exercise [Peri]:Total lymphocytes | 0.25 | [-1.27, 1.79] | 0.624 | 0.575 | 0.28 | 2675.3 | 1.000 |
| SNS index \| Time-to-exercise [Post]:Total lymphocytes | 0.03 | [-1.5, 1.57] | 0.515 | 0.463 | 0.26 | 2612.4 | 1.000 |
| SNS index \| Time-to-exercise [Peri]:B lymphocytes | -0.33 | [-0.83, 0.18] | 0.899 | 0.815 | 0.19 | 4224.3 | 1.000 |
| SNS index \| Time-to-exercise [Post]:B lymphocytes | -0.03 | [-0.52, 0.5] | 0.538 | 0.388 | 0.09 | 4279.1 | 1.000 |
| SNS index \| Time-to-exercise [Peri]:T lymphocytes | -0.27 | [-1.74, 1.2] | 0.632 | 0.585 | 0.28 | 2884.0 | 1.000 |
| SNS index \| Time-to-exercise [Post]:T lymphocytes | 0.13 | [-1.31, 1.63] | 0.567 | 0.518 | 0.27 | 2765.5 | 1.000 |
| SNS index \| Time-to-exercise [Peri]:T CD4+ lymphocytes | 0.05 | [-0.86, 0.93] | 0.547 | 0.461 | 0.16 | 3372.7 | 1.001 |
| SNS index \| Time-to-exercise [Post]:T CD4+ lymphocytes | -0.13 | [-1.05, 0.75] | 0.608 | 0.520 | 0.16 | 3261.3 | 1.000 |
| SNS index \| Time-to-exercise [Peri]:T CD8+ lymphocytes | -0.11 | [-0.73, 0.51] | 0.632 | 0.508 | 0.11 | 3937.5 | 1.000 |
| SNS index \| Time-to-exercise [Post]:T CD8+ lymphocytes | -0.14 | [-0.78, 0.45] | 0.671 | 0.548 | 0.11 | 3951.0 | 1.000 |
| SNS index \| Time-to-exercise [Peri]:NK lymphocytes | 0.19 | [-0.51, 0.93] | 0.704 | 0.600 | 0.14 | 2160.8 | 1.000 |
| SNS index \| Time-to-exercise [Post]:NK lymphocytes | 0.02 | [-0.7, 0.74] | 0.519 | 0.411 | 0.12 | 2173.2 | 1.000 |
| SNS index \| Time-to-exercise [Peri]:CD4+/CD8+ ratio | -0.21 | [-0.61, 0.21] | 0.848 | 0.709 | 0.12 | 5350.7 | 1.000 |
| SNS index \| Time-to-exercise [Post]:CD4+/CD8+ ratio | -0.05 | [-0.45, 0.34] | 0.600 | 0.409 | 0.07 | 5271.3 | 1.000 |
| SNS index \| Time-to-exercise [Peri]:CD56^bright NK lymphocytes | -0.12 | [-0.65, 0.41] | 0.660 | 0.518 | 0.10 | 2845.0 | 1.000 |
| SNS index \| Time-to-exercise [Post]:CD56^bright NK lymphocytes | 0.00 | [-0.54, 0.52] | 0.499 | 0.352 | 0.09 | 2805.8 | 1.000 |
| SNS index \| Time-to-exercise [Peri]:CD56^dim NK lymphocytes | 0.32 | [-0.12, 0.78] | 0.924 | 0.836 | 0.20 | 2720.4 | 1.000 |
| SNS index \| Time-to-exercise [Post]:CD56^dim NK lymphocytes | 0.04 | [-0.41, 0.49] | 0.570 | 0.400 | 0.08 | 2638.2 | 1.000 |
| Stress index \| Time-to-exercise [Peri] | 1.26 | [1.01, 1.51] | 1.000 | 1.000 | 6974.02 | 10277.6 | 1.000 |
| Stress index \| Time-to-exercise [Post] | -0.08 | [-0.33, 0.18] | 0.722 | 0.428 | 0.05 | 10680.2 | 1.000 |
| Stress index \| Total lymphocytes | -0.56 | [-1.85, 0.75] | 0.800 | 0.755 | 0.32 | 2504.9 | 1.000 |
| Stress index \| B lymphocytes | 0.02 | [-0.44, 0.44] | 0.527 | 0.352 | 0.07 | 3837.7 | 1.000 |
| Stress index \| T lymphocytes | 0.03 | [-1.27, 1.22] | 0.514 | 0.455 | 0.22 | 2536.2 | 1.000 |
| Stress index \| T CD4+ lymphocytes | 0.25 | [-0.53, 1.03] | 0.728 | 0.639 | 0.16 | 2690.7 | 1.000 |
| Stress index \| T CD8+ lymphocytes | 0.34 | [-0.2, 0.86] | 0.895 | 0.812 | 0.19 | 3178.4 | 1.000 |
| Stress index \| NK lymphocytes | 0.31 | [-0.3, 0.92] | 0.841 | 0.755 | 0.18 | 2139.5 | 1.000 |
| Stress index \| CD4+/CD8+ ratio | 0.14 | [-0.21, 0.5] | 0.784 | 0.590 | 0.08 | 4480.4 | 1.000 |
| Stress index \| CD56^bright NK lymphocytes | -0.03 | [-0.49, 0.4] | 0.559 | 0.386 | 0.08 | 2703.1 | 1.000 |
| Stress index \| CD56^dim NK lymphocytes | 0.17 | [-0.21, 0.56] | 0.811 | 0.646 | 0.10 | 2535.3 | 1.000 |
| Stress index \| Sex [Female] | -0.43 | [-0.94, 0.05] | 0.958 | 0.905 | 0.36 | 8358.1 | 1.001 |
| Stress index \| Age [+1 SD] | -0.08 | [-0.22, 0.06] | 0.878 | 0.404 | 0.05 | 9405.8 | 1.000 |
| Stress index \| Systolic BP [+1 SD] | 0.01 | [-0.13, 0.14] | 0.537 | 0.087 | 0.02 | 9169.6 | 1.000 |
| Stress index \| Diastolic BP [+1 SD] | -0.07 | [-0.23, 0.09] | 0.797 | 0.356 | 0.04 | 6610.4 | 1.000 |
| Stress index \| BMI [+1 SD] | -0.05 | [-0.32, 0.22] | 0.645 | 0.361 | 0.05 | 4610.9 | 1.000 |
| Stress index \| Total fat [+1 SD] | 0.15 | [-0.2, 0.5] | 0.812 | 0.624 | 0.09 | 4476.1 | 1.000 |
| Stress index \| Total muscle [+1 SD] | -0.06 | [-0.31, 0.21] | 0.666 | 0.368 | 0.05 | 5289.9 | 1.000 |
| Stress index \| (SFT) TMST [+1 SD] | 0.08 | [-0.09, 0.24] | 0.816 | 0.380 | 0.04 | 7973.1 | 1.000 |
| Stress index \| (SFT) TUG [+1 SD] | 0.08 | [-0.07, 0.23] | 0.860 | 0.412 | 0.05 | 7854.1 | 1.000 |
| Stress index \| (SFT) CSTS [+1 SD] | -0.07 | [-0.23, 0.08] | 0.823 | 0.377 | 0.04 | 8717.5 | 1.000 |
| Stress index \| Time-to-exercise [Peri]:Total lymphocytes | 0.70 | [-1.16, 2.45] | 0.777 | 0.744 | 0.41 | 2831.0 | 1.000 |
| Stress index \| Time-to-exercise [Post]:Total lymphocytes | 0.79 | [-1.01, 2.57] | 0.808 | 0.776 | 0.43 | 2759.1 | 1.000 |
| Stress index \| Time-to-exercise [Peri]:B lymphocytes | -0.56 | [-1.16, 0.04] | 0.967 | 0.937 | 0.57 | 4319.8 | 1.000 |
| Stress index \| Time-to-exercise [Post]:B lymphocytes | -0.19 | [-0.79, 0.41] | 0.729 | 0.609 | 0.12 | 4318.9 | 1.000 |
| Stress index \| Time-to-exercise [Peri]:T lymphocytes | -0.72 | [-2.46, 0.97] | 0.791 | 0.756 | 0.39 | 2991.3 | 1.000 |
| Stress index \| Time-to-exercise [Post]:T lymphocytes | -0.17 | [-1.9, 1.54] | 0.573 | 0.529 | 0.30 | 2849.7 | 1.001 |
| Stress index \| Time-to-exercise [Peri]:T CD4+ lymphocytes | 0.12 | [-0.94, 1.16] | 0.589 | 0.513 | 0.19 | 3384.1 | 1.000 |
| Stress index \| Time-to-exercise [Post]:T CD4+ lymphocytes | -0.37 | [-1.42, 0.69] | 0.754 | 0.692 | 0.23 | 3155.3 | 1.000 |
| Stress index \| Time-to-exercise [Peri]:T CD8+ lymphocytes | -0.11 | [-0.87, 0.6] | 0.620 | 0.514 | 0.13 | 3952.4 | 1.000 |
| Stress index \| Time-to-exercise [Post]:T CD8+ lymphocytes | -0.31 | [-1.05, 0.4] | 0.800 | 0.717 | 0.17 | 3766.3 | 1.000 |
| Stress index \| Time-to-exercise [Peri]:NK lymphocytes | 0.24 | [-0.62, 1.08] | 0.717 | 0.631 | 0.17 | 2318.6 | 1.000 |
| Stress index \| Time-to-exercise [Post]:NK lymphocytes | -0.22 | [-1.06, 0.62] | 0.701 | 0.615 | 0.16 | 2277.0 | 1.001 |
| Stress index \| Time-to-exercise [Peri]:CD4+/CD8+ ratio | -0.23 | [-0.71, 0.23] | 0.820 | 0.698 | 0.13 | 5302.2 | 1.000 |
| Stress index \| Time-to-exercise [Post]:CD4+/CD8+ ratio | -0.02 | [-0.48, 0.45] | 0.525 | 0.366 | 0.08 | 5167.3 | 1.000 |
| Stress index \| Time-to-exercise [Peri]:CD56^bright NK lymphocytes | -0.29 | [-0.9, 0.33] | 0.819 | 0.727 | 0.16 | 2969.6 | 1.000 |
| Stress index \| Time-to-exercise [Post]:CD56^bright NK lymphocytes | -0.01 | [-0.61, 0.65] | 0.509 | 0.387 | 0.10 | 2938.9 | 1.001 |
| Stress index \| Time-to-exercise [Peri]:CD56^dim NK lymphocytes | 0.45 | [-0.08, 0.97] | 0.955 | 0.907 | 0.36 | 2866.1 | 1.000 |
| Stress index \| Time-to-exercise [Post]:CD56^dim NK lymphocytes | 0.00 | [-0.52, 0.54] | 0.502 | 0.360 | 0.09 | 2715.7 | 1.001 |

# References

R Core Team (2021) *R: A language and environment for statistical computing*, Vienna, Austria: R Foundation for Statistical Computing. Available at: <https://www.R-project.org/>.

Van de Schoot R, Depaoli S, King R, Kramer B, Märtens K, Tadesse MG, Vannucci M, Gelman A, Veen D, Willemsen J & others (2021) Bayesian statistics and modelling. *Nature Reviews Methods Primers* 1, 1.
